# Neuronal miR-29a protects from obesity in adult mice

**DOI:** 10.1016/j.molmet.2022.101507

**Published:** 2022-04-29

**Authors:** Yuan Ma, Nicola Murgia, Yu Liu, Zixuan Li, Chaweewan Sirakawin, Ruslan Konovalov, Nikolai Kovzel, Yang Xu, Xuejia Kang, Anshul Tiwari, Patrick Malonza Mwangi, Donglei Sun, Holger Erfle, Witold Konopka, Qingxuan Lai, Syeda Sadia Najam, Ilya A. Vinnikov

**Affiliations:** 1 Laboratory of Molecular Neurobiology, Sheng Yushou Center of Cell Biology and Immunology, Department of Genetics and Developmental Biology, School of Life Sciences and Biotechnology, Shanghai Jiao Tong University, Shanghai, China; 2 Sheng Yushou Center of Cell Biology and Immunology, Department of Genetics and Developmental Biology, School of Life Sciences and Biotechnology, Shanghai Jiao Tong University, Shanghai, China; 3 Advanced Biological Screening Facility, BioQuant, University of Heidelberg, Heidelberg, Germany; 4 Laboratory of Neuroplasticity and Metabolism, Department of Life Sciences and Biotechnology, Łukasiewicz PORT Polish Center for Technology Development, Wrocław, Poland

**Keywords:** Obesity, microRNA, Neuron, Mice, Hypothalamus, A/P, anterio-posterior coordinates relative to Bregma, ARH, arcuate nucleus of the hypothalamus, AU, arbitrary units, AUC, area under the curve, CAG, strong synthetic promoter driving high levels of gene expression in mammalian expression vectors, Cas9 mice, B6;129-Gt (ROSA)26Sor^tm1(CAG-cas9^∗,^−EGFP)Fezh^/J mice, CSF, cerebrospinal fluid, DicerCKO mice, CamK^CreERT2^Dicer^fl/fl^mice, D/V, dorso-ventral coordinates relative to Bregma, HITS-CLIP, high-throughput sequencing of RNA isolated by crosslinking immunoprecipitation, KEGG, Kyoto Encyclopedia of Genes and Genomes, LNA, locked nucleic acid, M/L, medio-lateral coordinates relative to Bregma, mTOR, mammalian target of rapamycin, NPY, neuropeptide Y, PI3K, phosphatidylinositol-3-kinase, POMC, proopiomelanocortin, POMC^Cre^ mice, Tg (Pomc1-cre)16Lowl/J mice, pS6, phosphorylated S6 ribosomal protein, rAAV, recombinant adeno-associated viral vector, RER, respiratory exchange ratio, SEM, standard error of means, sgR, sgRNA, single guide CRISPR RNA, vg/mL, vector genome copies per mL, wk, week

## Abstract

**Objective:**

Obesity, a growing threat to the modern society, represents an imbalance of metabolic queues that normally signal to the arcuate hypothalamic nucleus, a critical brain region sensing and regulating energy homeostasis. This is achieved by various neurons many of which developmentally originate from the proopiomelanocortin (POMC)-expressing lineage. Within the mature neurons originating from this lineage, we aimed to identify non-coding genes in control of metabolic function in the adulthood.

**Methods:**

In this work, we used microRNA mimic delivery and POMC^Cre^-dependent CRISPR-Cas9 knock-out strategies in young or aged mice. Importantly, we also used CRISPR guides directing suicide cleavage of Cas9 to limit the off-target effects.

**Results:**

Here we found that mature neurons originating from the POMC lineage employ miR-29a to protect against insulin resistance obesity, hyperphagia, decreased energy expenditure and obesity. Moreover, we validated the miR-29 family as a prominent regulator of the PI3K-Akt-mTOR pathway. Within the latter, we identified a direct target of miR-29a-3p, *Nras*, which was up-regulated in those and only those mature POMC^Cre^Cas9 neurons that were effectively transduced by anti-miR-29 CRISPR-equipped construct. Moreover, POMC^Cre^-dependent co-deletion of *Nras* in mature neurons attenuated miR-29 depletion-induced obesity.

**Conclusions:**

Thus, the first to our knowledge case of *in situ* Cre-dependent CRISPR-Cas9-mediated knock-out of microRNAs in a specific hypothalamic neuronal population helped us to decipher a critical metabolic circuit in adult mice. This work significantly extends our understanding about the involvement of neuronal microRNAs in homeostatic regulation.

## Highlights

• Delivery of miR-29a-3p to the arcuate hypothalamic nucleus attenuates obesity.
• Knock-out of genes in mature neurons by Cre-dependent CRISPR/Cas9 technique involving Cas9-cleaving sgRNAs to limit off-target effects.
• Deletion of miR-29a in mature Pomc^Cre^ neurons leads to early-onset insulin resistance and later to hyperphagia and decreased energy expenditure.
• POMC^Cre^-restricted deletion of miR-29a causes cell-autonomous *Nras* up-regulation leading to obesity.
• POMC^Cre^-restricted knock-out of *Nras*, a direct target of miR-29a-3p, attenuates obesity in mice.

## Graphical abstract

**Figure:**
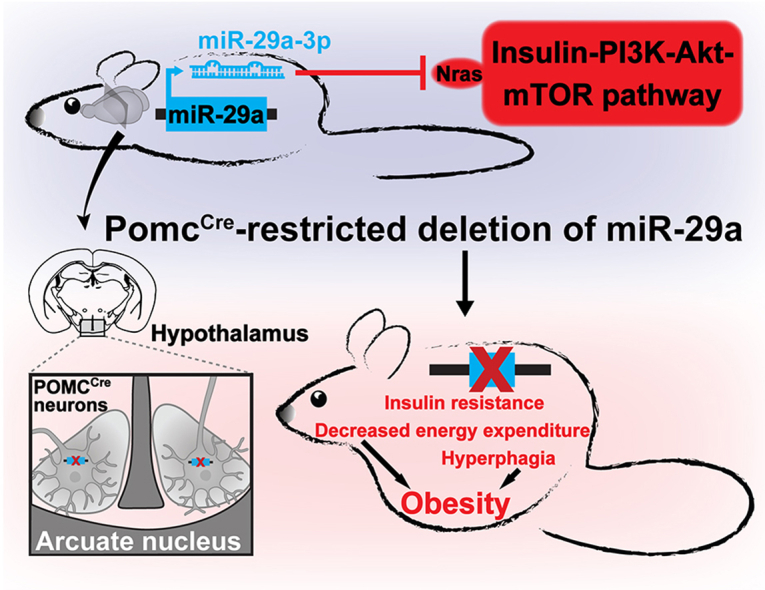

## 1 Introduction

Overweight and obesity are the most important modifiable risk factors promoting the global diabetes prevalence. The latter is projected to increase by 50% in 25 years from today's half a billion people living with diabetes worldwide [1]. The critical brain region for the regulation of energy homeostasis, the arcuate hypothalamic nucleus (ARH), comprises various types of cells mediating metabolic functions, such as the melanocortin system [2]. Within the latter, the two major neuronal populations express bioactive peptides of opposing metabolic roles. On the one pole, the appetite-inducing (orexigenic) neuronal population expresses Gad67, agouti-related protein and neuropeptide Y (NPY). On the other – the appetite-suppressing (anorexigenic) neuronal population expresses Tbx3 [3] transcription factor and proopiomelanocortin (POMC), the precursor of α-melanocyte-stimulating hormone [4]. These neurons signal to various hypothalamic and extra-hypothalamic structures to control food intake and energy expenditure [5]. Reporter analyses in Tg (Pomc1-cre)16Lowl/J (JAX: 005965, further referred to as POMC^Cre^) transgenic mouse line [6] revealed that all of the POMC-expressing precursor cells continue to express *Tbx3* throughout the adulthood [3]*.* However, half of them switch off the *Pomc* promoter during maturation with up to 17% neurons adopting the NPY-expressing identity [7,8]. Indeed, in the adulthood, many neuronal populations reveal traces of the POMC lineage, including 25–56% of mature NPY neurons [8,9] and 18% of fertility-regulating and nutrition-responsive *Kiss1*/*Tac2* neurons [9]. In mice, POMC^Cre^-dependent over-activation of the phosphatidylinositol-3-kinase (PI3K)-Akt-mammalian target of rapamycin (mTOR) pathway causes hyperpolarization of POMC neurons leading to obesity [[10], [11], [12]].

Previously, we and others discovered that expression of a critical nuclease for microRNA maturation, Dicer, in adult ARH [13] and developing POMC neurons [14,15], is critical for energy homeostasis. Furthermore, we identified a group of candidate microRNAs including miR-103–3p preventing PI3K-Akt-mTOR pathway over-activation and thus protecting against hyperphagic obesity induced by *Dicer1* deletion in the mouse hypothalamus [13]. Next, Croizier et al. demonstrated that the loss of miR-103–3p is critical for proper maturation of POMC neurons resulting in impaired glucose homeostasis due to this developmental defect [15]. However, no studies have explored the metabolic roles of specific microRNAs in defined cellular populations within the mature hypothalamus. Recently, using an *in vivo* reductionist approach, we have identified miR-29a and miR-15a within the arcuate hypothalamic nucleus as putative targets in Dicer deletion-induced obesity (Murgia et al., submitted) and confirmed the metabolic role of the latter microRNA in mature POMC lineage-derived neurons.

Here, we demonstrate the obesity-suppressive function of miR-29a in this mature neuronal population. Importantly, *in vitro* and *in vivo* experiments revealed *Nras*, a highly conserved GTPase and a potent activator of the PI3K-Akt pathway [16], as a direct target of miR-29a-3p.

## 2 Results

### 2.1 MiR-29a prevents PI3K-Akt-mTor up-regulation and its delivery to the arcuate nucleus attenuates obesity in adult mice

**Figure 1:**
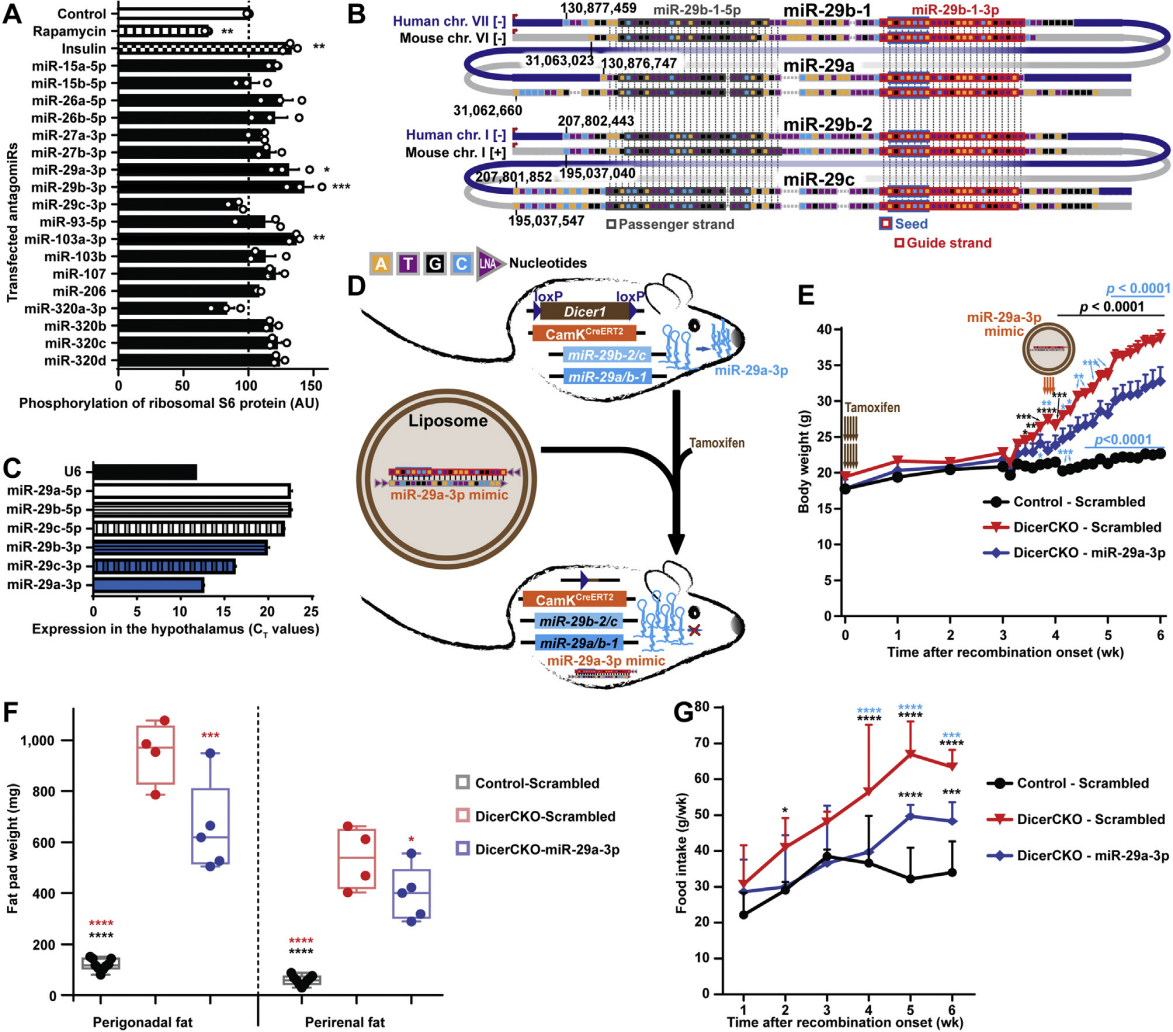
**MiR-29a prevents PI3K-Akt-mTORup-regulation and and its delivery to the arcuate nucleus attenuates obesity in adult mice.** (A) Quantification of pS6 signal in HeLa cells transfected with indicated human microRNA inhibitors or treated with 300 nM insulin or 500 nM rapamycin (n = 3). (B) Scheme illustrating phylogenetic conservation of the miR-29 family. Both clusters on each of the chromosomes (chr.) in mice and humans are aligned to each other, transcription direction is shown by a red arrow on the left, genetic positions are indicated. (C) TaqMan qPCR analysis of mature forms of guide (bottom) and passenger (top) strands of the miR-29 family in the wild type mouse hypothalamus compared to U6 RNA (n = 4). (D–G) MicroRNA mimic injection to DicerCKO mice with the scheme of the experiment (D), body weight (E), fat pad weights (F) and food intake (G) analyses. Liposomal formulation of miR-29a-3p mimics was injected bilaterally to the arcuate hypothalamic nucleus of adult DicerCKO female mice on the 4th week after tamoxifen treatment. n = 9, 4 and 5 for Control-Scrambled (C–S), DicerCKO-Scrambled (D–S) and DicerCKO-miR-29a-3p mimic (D-29) groups, respectively. Error bars represent SEM. ∗, *p* < 0.05; ∗∗, *p* < 0.01; ∗∗∗, *p* < 0.001; ∗∗∗∗, *p* < 0.0001 as assessed by 1-way (A,F) or 2-way (E,G) ANOVA followed by post-hoc Holm–Sidak tests with significances indicated vs. the control group or vs. the groups outlined with a respective color.

To analyze the potential of previously identified candidate microRNAs [13] to inhibit the PI3K-Akt-mTOR pathway, we first quantified phosphorylation of its downstream target, S6 ribosomal protein (pS6) in HeLa cells after transfection of antagomiRs targeting these microRNA families in extremely low insulin conditions. Out of all microRNA groups, we found that depletion of only miR-29b-3p, miR-103a-3p and miR-29a-3p leads to a significant increase in pS6 signal (Figure 1A, Table S1). These results suggest that along with miR-103a-3p that we previously identified using very stringent selection criteria [13], the miR-29 family might also prevent the PI3K-Akt-mTOR pathway over-activation. This highly conserved family is encoded by two double-microRNA clusters both in humans and mice (Figure 1B). Expression of this family in the peripheral tissues had been previously found to be largely detrimental for glucose and lipid metabolism [[17], [18], [19], [20], [21], [22]], however its role in the regulation of energy homeostasis in the central nervous system remains unknown. We found that these microRNAs are highly abundant in the mouse hypothalamus (Figure 1C) with miR-29a-3p drastically increasing the expression in the first postnatal weeks [23], reaching the levels of house-keeping genes in adult mice (Figure 1C). Notably, miR-29a-3p is also highly abundant in the POMC lineage-derived neurons and other cells within ARH of adult mice (Figure S1, Table S1).

To prove that miR-29a-3p contributes to energy balance control *in vivo*, we aimed to attenuate *Dicer1* depletion-induced obesity in adult CamK^CreERT2^Dicer^fl/fl^ [13] (further referred to as DicerCKO) mice by injecting a liposomal formulation of locked nucleic acid (LNA)-stabilized mimics [27] of miR-29a-3p into ARH (Tables S1–2 and Figure 1D). Compared to DicerCKO animals injected with scrambled oligonucleotides, mimic-injected DicerCKO mice had significantly attenuated body and fat pad weights, as well as food intake (Figure 1E–G). These results indicate i). that, when delivered in the adulthood, miR-29a-3p is capable to attenuate hyperphagic obesity and ii). that DicerCKO-associated phenotype is mediated (at least partially) by this microRNA.

### 2.2 Loss of miR-29a in the adult mouse arcuate hypothalamic nucleus leads to obesity

**Figure 2:**
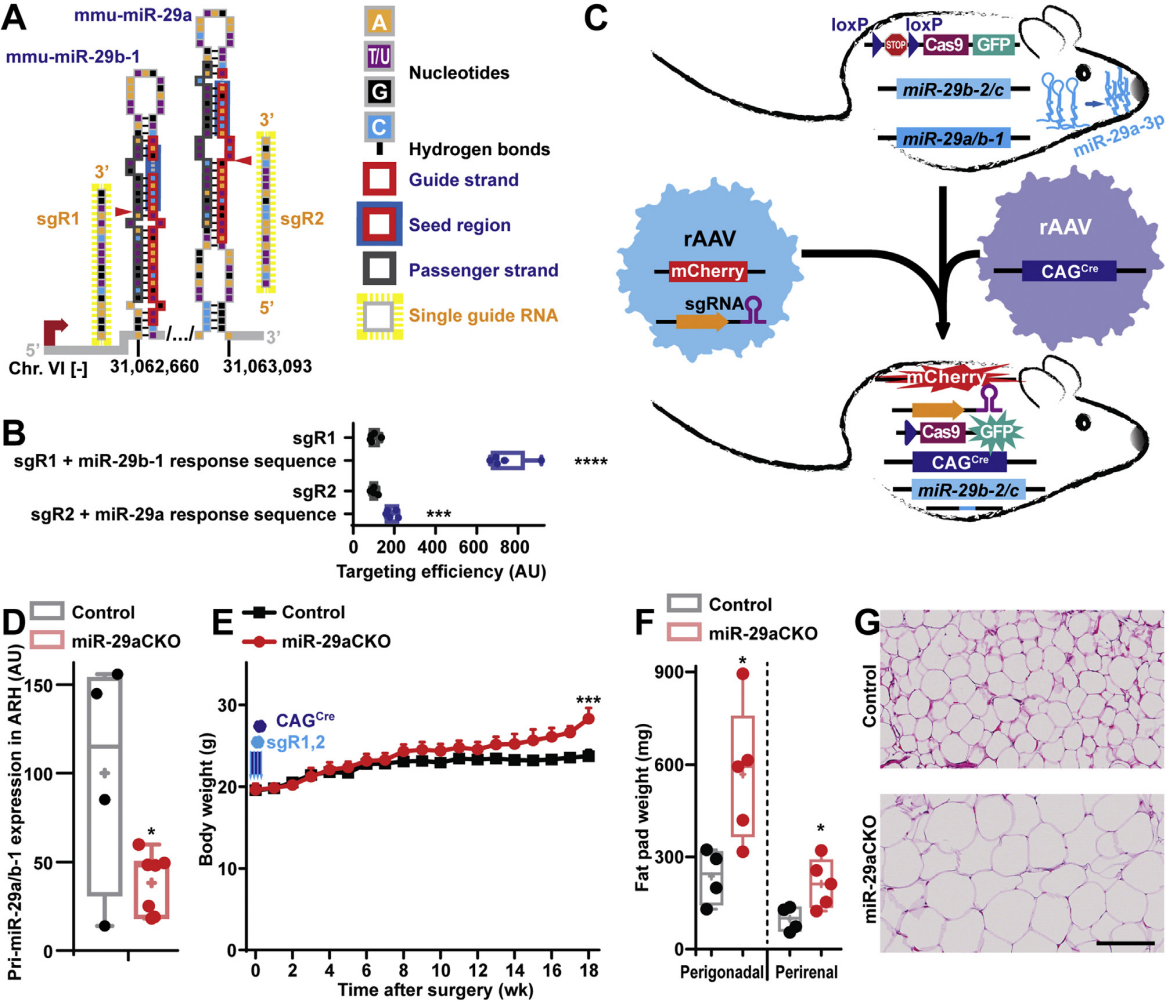
**Loss of miR-29a in the arcuate hypothalamic nucleus during adulthood leads to obesity in mice.** (A) Scheme representing a genetic context of the mmu-miR-29a/b-1 cluster and the single guide RNAs (sgR) targeting it. The cutting positions are indicated by red triangles. (B) On-target effectivity of sgR-1 and -2 in HEK293T cell line by split luciferase assay (n = 5). (C) Scheme of the experiment to knock-out the miR-29a/b-1 cluster in the arcuate hypothalamic nucleus (ARH) of adult mice. (D) qPCR analysis of pri-miR-29a/b-1 in the microdissected ARH of miR-29aCKO mice with β-actin used as a reference gene. (E–G) Body weight (E), fat pad weights (F) and microphotographs of hematoxylin and eosin-stained perigonadal adipose tissue (G) in adult miR-29aCKO mice bilaterally injected into 2 coronal planes of ARH by a mixture of adeno-associated viral vectors (rAAVs) equipped with single guide RNAs- and CAG-Cre. n = 7 and 4 (D), 5 and 7 for (E), 5 and 4 for (F) for miR-29aCKO and controls, respectively). Error bars represent SEM. ∗, *p* < 0.05; ∗∗∗, *p* < 0.001; ∗∗∗∗, *p* < 0.0001 as assessed by unpaired two-tailed Student's t-test (B, D, F) or 2-way ANOVA followed by post-hoc Holm–Sidak pairwise comparison tests (E). Scale bar (μm): 125.

To further investigate the role of miR-29a in energy homeostasis control, we used CRISPR-Cas9 system to knock-out the miR-29a/b-1 cluster in ARH of the adult animals (further referred to as miR-29aCKO mice) (Figure 2A). First, we predicted and validated single guide RNAs (sgRs) targeting this cluster located on the chromosome VI (Figure 2A, Table S1 and File S1). Potential off-target sites were predicted by https://chopchop.cbu.uib.no/ software and analyzed *in vitro* and *in vivo* (File S1). Then, we subcloned and packaged double-sgR cassette under RNA polymerase III-specific promoters within an adeno-associated virus vector (rAAV) co-expressing mCherry fluorescent protein (Figure 2C, Table S1 and File S2). Stereotaxic injection (Table S2) of this vector mixed with rAAV expressing CAG promoter-driven Cre [13] into ARH of adult B6;129-Gt (ROSA)26Sor^tm1(CAG-cas9^^∗^^,−EGFP)Fezh^/J (JAX: 024857, further referred to as Cas9) mice [28] resulted in a significant down-regulation of miR-29a-3p expression in ARH with mild and transient, but significant weight gain in male miR-29aCKO mice, which was not accompanied by food intake, fat pad weight or body temperature changes (Figure 2D, Figure S2A-D**)**. Interestingly, female miR-29aCKO mice exhibited a late onset weight gain and fat tissue hypertrophy that were also not accompanied by food intake or body temperature changes during the early stages of this phenotype (Figure 2E–G and Figure S2E-F). These data indicate that expression of miR-29a in ARH throughout the adulthood is critical for energy homeostasis.

### 2.3 Knock-out of miR-29a in young and aged POMC^Cre^Cas9 mice leads to obesity

**Figure 3:**
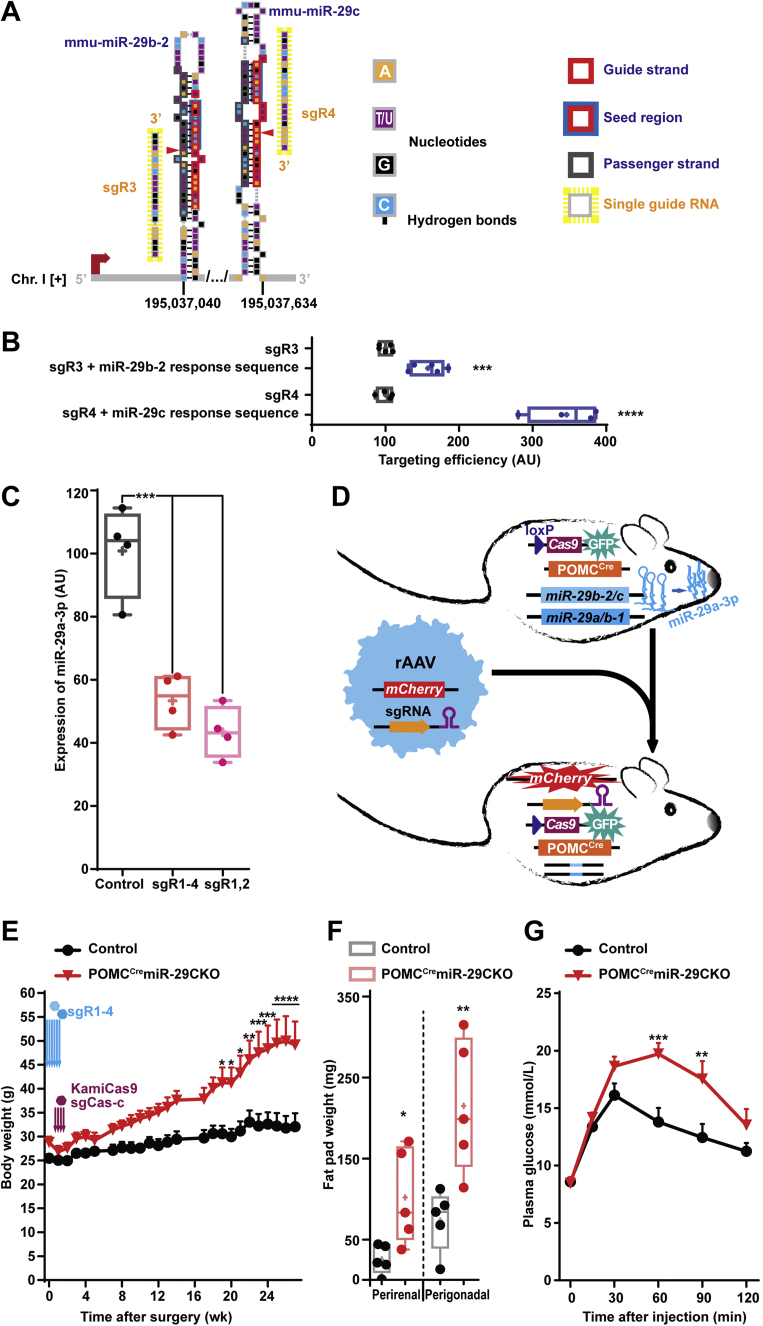
**POMC^Cre^-restricted knock-out of the miR-29 family leads to obesity in aged mice.** (A) Scheme representing a genetic context of the mmu-miR-29b-2/c cluster and the single guide RNAs (sgRs) targeting it. (B) On-target effectivity of sgR-3 and -4 targeting miR-29b-2 and miR-29c, respectively, in HEK293T cell line by split luciferase assay (n = 5). (C) TaqMan qPCR analysis of U6-normalized miR-29a-3p expression in FACS-sorted HT-22 mouse hippocampal cell line transfected by vectors equipped with sgRs targeting one or both clusters (n = 4). (D) Scheme of the experiment to knock-out both miR-29 clusters in mature POMC-lineage derived neurons. (E–G) Body weight (E), fat pad weights (F) and glucose tolerance test (G, 20 weeks after surgery) in 23-week-old POMC^Cre^Cas9 female mice (referred to as POMC^Cre^miR-29CKO) bilaterally injected with rAAVs equipped with sgRs targeting both miR-29 clusters and 1 week later injected with rAAVs equipped with two *Cas9* gene-targeting sgRNAs (n = 5). Error bars represent SEM. ∗, *p* < 0.05; ∗∗, *p* < 0.01; ∗∗∗, *p* < 0.001; ∗∗∗∗, *p* < 0.0001 as assessed by unpaired two-tailed Student's t-test (B,F), 1-way (C) or 2-way (E,G) ANOVA followed by post-hoc Holm–Sidak pairwise comparison tests.

Next, we intended to narrow down the scope of neuronal populations involved in the observed age-related phenotype (Figure 3D). Previously, we showed that *Dicer1* deletion-associated hyperphagic obesity is strongly dependent on over-activation of the PI3K-Akt-mTOR pathway [13]. Interestingly, several research groups demonstrated that POMC^Cre^-restricted over-activation of this pathway causes K_ATP_ channels-associated hyperpolarization of POMC neurons. This in turn leads to age-dependent hyperphagic obesity [[10], [11], [12]]. We thus hypothesized that POMC^Cre^-restricted depletion of the miR-29 family can lead to age-related obesity in animals further referred to as POMC^Cre^miR-29aCKO mice. After prediction and validation of two sgRs targeting miR-29a/b-1 cluster and two sgRs targeting miR-29b-2/c clusters, we stereotaxically injected both rAAV vectors each expressing those double-sgR cassettes into ARH of 23-week-old POMC^Cre^Cas9 female mice (Figure 1B, Figures 2A and 3A-D and Tables S1–S2, Files S1-S2). Notably, one week after stereotaxic injection, we delivered into ARH of POMC^Cre^miR-29CKO mice another rAAV vector equipped with sgRs designed to target *Cas9* [29,30] to restrain the potential off-target effects of the CRISPR-Cas9 system. In addition to qRT-PCR (Figure 2D), *in vivo* knock-out by the CRISPR-Cas9 system was validated by miR-29a-3p *in situ* hybridization (Fig. S3B). Interestingly, conditional knock-out of all miR-29 family members in aged female POMC^Cre^miR-29CKO mice resulted in late onset obesity, fat pad enlargement and insulin resistance (Figure 3F–H). Similarly, a conditional knock-out of miR-29 family in 21-week-old POMC^Cre^miR-29CKO males followed by inactivation of Cas9 resulted in a mild and transient, but significant weight increase (Fig. S3).

**Figure 4:**
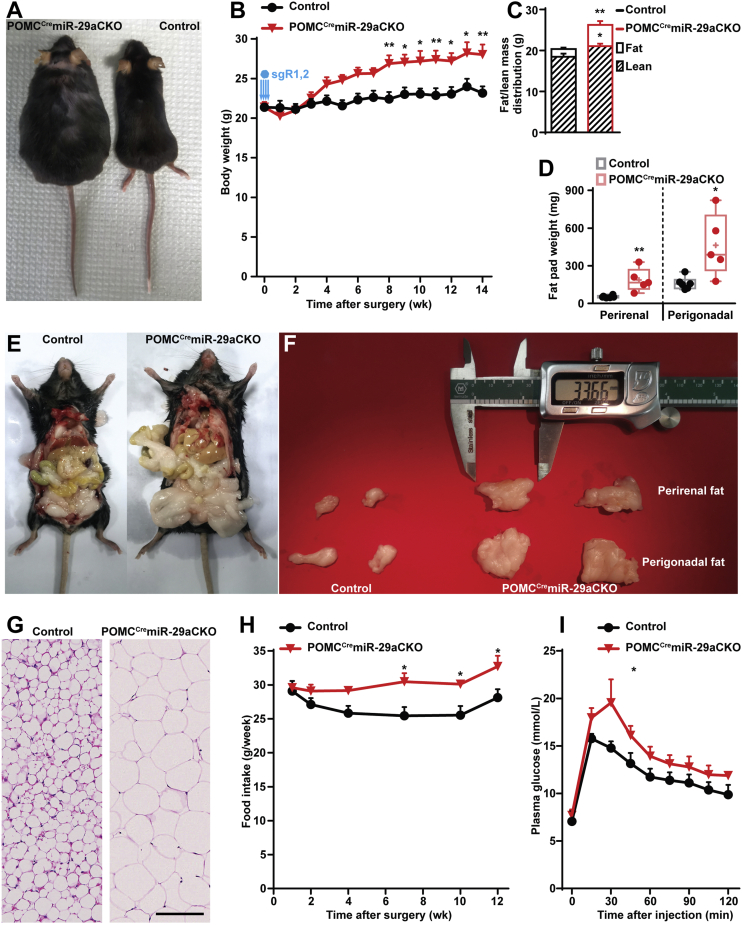
**Knock-out of miR-29a in POMC^Cre^Cas9 mice leads to obesity and insulin resistance.** (A–I) Phenotypic analyses of POMC^Cre^Cas9 females bilaterally injected by rAAVs equipped with sgRNAs targeting miR-29a/b-1 cluster with body macrophotographs (A), body weight (B) and Echo-MRI-based body composition analysis (C). For fat tissue, fat pad weights (D) were analyzed, macro- (E,F) and microphotographs of perigonadal (D-F,G) and perirenal (D–F) adipose tissue were taken. Food intake analysis (H) and glucose tolerance test 14 weeks after surgery (I). n = 5 and 6 for POMC^Cre^miR-29aCKO and control mice, respectively). Error bars represent SEM. ∗, *p* < 0.05; ∗∗, *p* < 0.01; ∗∗∗, *p* < 0.001; ∗∗∗∗, *p* < 0.0001 as assessed by unpaired two-tailed Student's t-test (C,D) or 2-way ANOVA followed by post-hoc Holm–Sidak pairwise comparison tests (B,H,I). Scale bar (μm): 125.

Strikingly, just 4 weeks after surgery, several weeks before the onset of the weight phenotype (Fig. S3F), young POMC^Cre^miR-29CKO mice demonstrated impaired insulin sensitivity (Fig. S3G). To examine whether a conditional knock-out of miR-29a/b-1 cluster alone is sufficient to induce obesity and to confirm whether this phenotype is independent of age, we stereotaxically infected ARH of young POMC^Cre^Cas9 mice with rAAV expressing sgR-double cassette targeting this cluster (Figure 2A–B, 3C, Tables S1–2, Files S1-2). Indeed, mutant POMC^Cre^miR-29aCKO females exhibited hyperphagic obesity accompanied by fat tissue hypertrophy and insulin resistance (Figure 3F–H, 4) indicating that miR-29a is critical for normal metabolic balance and is independent of age. In accordance with the previously observed sex-dependent phenotypes (Figs. S2 and S4), conditional inactivation of the miR-29 family in young POMC^Cre^miR-29aCKO males failed to induce weight gain in chow diet conditions within 10 weeks after infection, unless stimulated by high fat diet (Fig. S3D). Interestingly, mild hyperphagia accompanying obesity phenotype in POMC^Cre^miR-29aCKO mice might indicate the existence of yet another mechanism additionally contributing to the weight gain. Indeed, POMC^Cre^miR-29aCKO mice demonstrated decreased energy expenditure during the activity phase, as indicated by a decline in oxygen consumption and locomotor activity during the subjective night (Fig. S4).

### 2.4 Nras, a direct target of miR-29a-3p, mediates the metabolic phenotype in the adult mice

**Figure 5:**
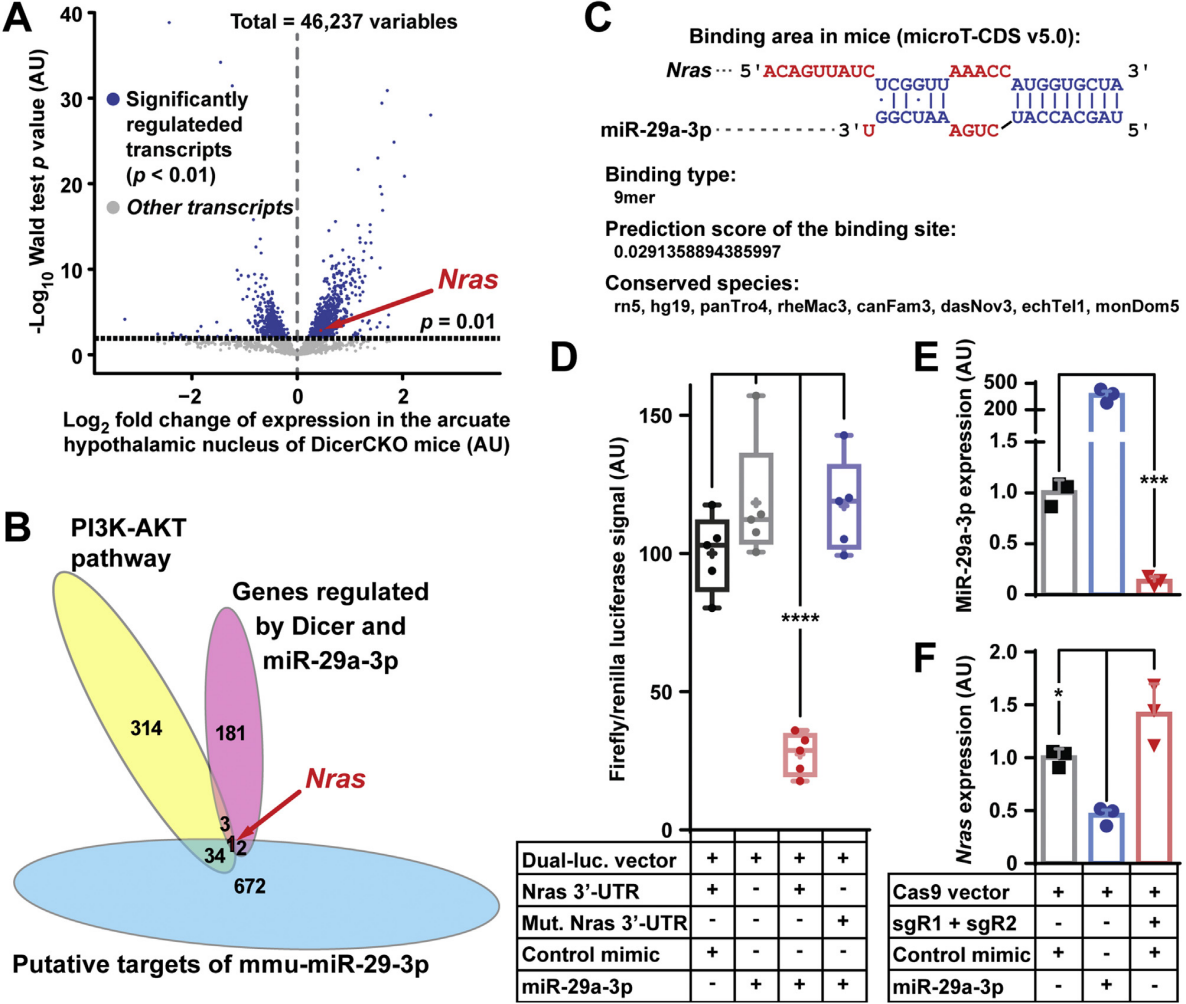
**Nras is a target of miR-29a-3p.** (A) Volcano plot demonstrating *Nras* significantly up-regulated in the arcuate hypothalamic nucleus ARH of DicerCKO-Scrambled mice compared to Control-Scrambled mice (n = 3). (B) Euler diagram identifies *Nras* as the candidate target gene with the indicated criteria. (C,D) Prediction by Diana-microT-CDS [31] (C) and validation by dual luciferase assay (D) of a highly phylogenetically conserved binding site for miR-29a-3p in the mouse *Nras* 3′-UTR. (E,F) Functional validation of *Nras* targeting *in vitro* assessed by qPCR analyses of expression of miR-29a-3p (E) and *Nras* (F) upon transfection of HT-22 cells with miR-29a-3p mimic or SpCas9-expressing vector equipped with sgR-1 and -2 targeting miR-29a/b-1 cluster (n = 3). U6 and β-actin were used as the reference genes for normalization. Error bars represent SEM. ∗, *p* < 0.05; ∗∗∗, *p* < 0.001; ∗∗∗∗, *p* < 0.0001 as assessed by Wald test (A) 1-way ANOVA followed by post-hoc Holm–Sidak pairwise comparison test (D,F) and unpaired two-tailed Student's t-test (E).

According to our data, miR-29 family effectively suppresses PI3K-Akt-mTOR pathway *in vitro* (Figure 1A). Since POMC^Cre^-restricted over-activation of this pathway is associated with obesity [10,11], we sought to investigate putative miR-29a-3p targets mediating a protective metabolic effect of this microRNA. Among multiple candidates, we identified *Nras*, the only gene in the PI3K-Akt pathway (https://www.kegg.jp/) that, i). was significantly up-regulated in microdissected ARH tissue of DicerCKO-Scrambled compared to Control-Scrambled mice, ii). was significantly down-regulated in ARH of DicerCKO-miR-29a-3p compared to DicerCKO-Scrambled mice (Figures 1D and 5A,B, Figure S5, File S3A), and iii). was predicted by Diana microT-CDS [31] as a target of miR-29a and other miR-29 family members (Table S3). Notably, genome-wide association studies had identified that the rs7549358 mutation in NRAS associates with body mass index in humans [32] suggesting its potential involvement in regulation of energy homeostasis in mammals. Various prediction tools including microT-CDS identified Nras, a highly conserved (Fig. S5B) [33] GTPase and a potent activator of the PI3K-Akt pathway [16,34], as a target of miR-29 family members in humans and mice (Figure 5C, Table S3).

Further, using miR-29a-3p or control mimics co-transfected with dual-luciferase vector expressing intact or miR-29a binding site-mutated *Nras* 3′-UTR fragment, we successfully validated the only highly conserved binding site of this microRNA (Table S1, Figure 5C,D). Interestingly, using a different technique, high-throughput sequencing of RNA isolated by crosslinking immunoprecipitation (HITS-CLIP), the same binding site within *Nras* had been previously independently validated in the bone marrow tissue as critical for binding to miR-29a-3p [35]. To further validate this regulatory mechanism in mouse neuronal cell line, we co-transfected immortalized mouse hippocampal cell line HT-22 with miR-29a-3p or control mimics and SpCas9-equipped vectors co-expressing sgR cassettes targeting the miR-29a/b-1 cluster or an empty sgR cassette (Figure 2A, Table S1 and File S2). Transfection of miR-29a-3p mimic strongly suppressed *Nras* expression while knock-out of this microRNA led to a significant up-regulation of this gene *in vitro* (Figure 5E–F).

**Figure 6:**
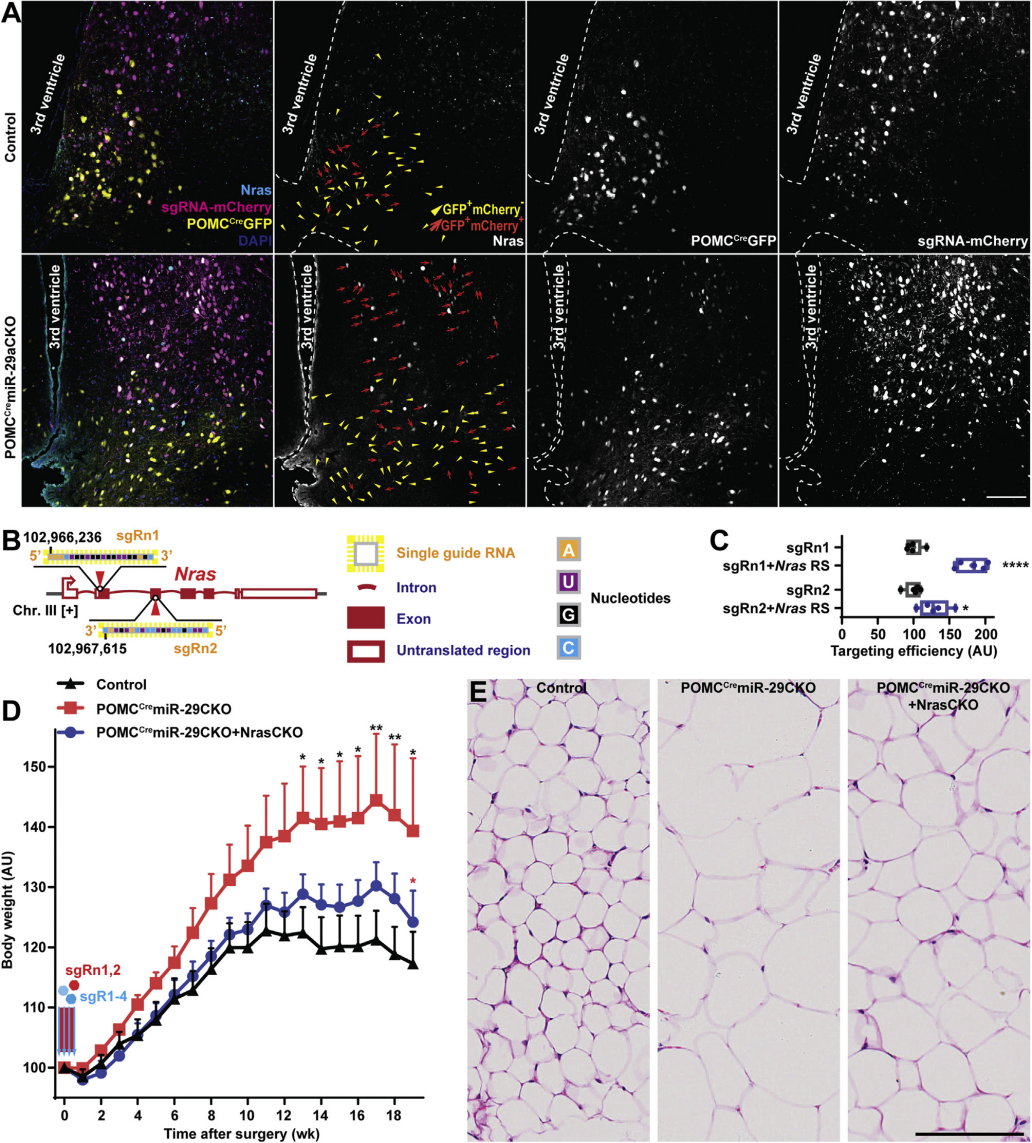
**Knock-out of Nras attenuates obesity in POMC^Cre^miR-29CKO mice.** (A) Confocal microscopy and co-localization studies reveal that Nras is expressed only in those POMC-lineage derived cells that are effectively transduced by miR-29a/b-1 cluster-targeting rAAV (POMC^Cre^GFP ^+^ mCherry ^+^ cells are indicated by red arrows), but not in non-targeted POMC^Cre^GFP ^+^ mCherry^−^ cells indicated by yellow triangles or POMC^Cre^GFP ^+^ cells (triangles and arrows) in the group injected by non-targeting sgRNAs (upper row). (B) Scheme outlining a genetic context of the *Nras* gene and sgRn-1 and sgRn-2 targeting it with the cutting positions indicated by red triangles. (C) On-target effectivity analysis of these sgRs in HEK293T cell line assessed by split luciferase assay (n = 5). RS, response sequence. (D,E) Body weight analysis (D) and microphotographs of hematoxylin and eosin-stained perigonadal adipose tissue (E) in POMC^Cre^Cas9 male mice stereotaxically injected with rAAVs equipped with sgR1-4 (referred to as POMC^Cre^miR-29CKO), sgR1-4 and sgRn1,2 (referred to as POMC^Cre^miR-29CKO + NrasCKO) or controls (n = 7, 9, 5 respectively). Error bars represent SEM. ∗, *p* < 0.05; ∗∗, *p* < 0.01; ∗∗∗∗, *p* < 0.0001 as assessed by unpaired two-tailed Student's t-test (C) or 2-way ANOVA followed by post-hoc Holm–Sidak pairwise comparison test vs. the groups outlined with a respective color (D). Scale bars (μm): 100 (A), 125 (E).

To validate this interaction *in vivo*, we performed confocal co-localization studies of Nras immunofluorescence, mCherry autofluorescence as an indicator of an effective infection by sgR-equipped rAAV and GFP autofluorescence as a label of POMC^Cre^Cas9-expressing neurons in POMC^Cre^miR-29aCKO mice. Strikingly, while intact POMC^Cre^GFP^+^ mCherry^−^ neurons in the mutant group as well as all POMC^Cre^GFP^+^ neurons in the control group transduced with non-targeting sgRNAs kept Nras at undetectable levels, those and only those neurons that were successfully infected with rAAV equipped with miR-29-targeting sgRs (POMC^Cre^GFP^+^ mCherry^+^), exhibited up-regulation of Nras (Figure 6A). These results confirm targeting of *Nras* by neuronal miR-29a, in accordance with previously reported data on bone marrow tissues [35].

To prove that *Nras* plays a role in the obesity phenotype of POMC^Cre^miR-29CKO mice, we designed (Figure 6B, Table S1, File S1), validated (Figure 6C) and packaged *Nras*-targeting sgR-n1 and -n2 into rAAV (File S2). In our previous experiments (Figs. S2 and S3), we observed that the extent of the metabolic phenotypes depended on sex, with a relatively late onset of transient weight phenotype in ARH-restricted knock-out males. However, on a chow diet, we did not monitor metabolic parameters in POMC^Cre^miR-29CKO males beyond the 10th week after transduction. To test the possibility of the late onset phenotype, we injected 10-week-old POMC^Cre^Cas9 males with miR-29 family-targeting rAAVs into ARH. In addition to the control group, we have injected a group of age-matched POMC^Cre^Cas9 mice with sgR-equipped rAAVs targeting both miR-29 and *Nras* (Figure 6D). Indeed, POMC^Cre^miR-29CKO mice revealed early onset food intake increase, late onset obesity, hypertrophy of adipocytes, and increased insulin resistance (Figure 6D, Figure S5) while simultaneous deletion of *Nras* in the same type of neurons of POMC^Cre^miR-29CKO + NrasCKO mice significantly attenuated this phenotype indicating the contribution of Nras up-regulation to POMC^Cre^miR-29CKO phenotype.

## 3 Discussion

This is one of the first studies to mechanistically dissect a critical metabolic role of a single microRNA in a mature neuronal lineage within the hypothalamus. Here, we also address a long-standing question about the specific microRNAs mediating Dicer depletion-associated hypothalamic obesity. Indeed, the POMC^Cre^-dependent CRISPR-Cas9 technique used in this work demonstrates that miR-29a is critical for normal energy homeostasis control in the adulthood. Inactivation of this microRNA in adult POMC^Cre^miR-29aCKO mice leads to cell-autonomous up-regulation of Nras expression and, possibly, other regulators of the insulin-PI3K-Akt pathway within the first order melanocortinergic neurons. Loss of insulin sensitivity as early as 4 weeks after sgRNA transduction, the earliest phenotype in POMC^Cre^-dependent miR-29 knock-out mice, may lead to metabolic imbalance in the periphery. At the same time, cell-autonomous dysregulation of the Nras-insulin-PI3K-Akt axis within the POMC neurons may contribute to chronic impairment of anorexigenic signaling to the secondary regions culminating in the onset of hyperphagia and obesity. The latter is more pronounced in females and is accompanied by hypertrophy of adipocytes. This phenotype appears to be driven by both hyperphagia and decreased energy expenditure and can be attenuated *in vivo* by POMC^Cre^-dependent depletion of miR-29a′s target *Nras* in the same neurons.

According to our research logic, we aimed to gradually increase specificity of microRNAs and narrow down the neuronal populations associated with the obesity phenotype by using the following *in vivo* models in the following order: i). knock-out of Dicer in the forebrain [13] ⇨ ii). knock-out of Dicer in the arcuate nucleus neurons [13] ⇨ iii). delivery of mixture of 10 microRNA mimics to the arcuate hypothalamic nucleus [13] ⇨ iv). delivery of miR-29a-3p mimic to the arcuate nucleus neurons (Figure 1) ⇨ v). knock-out of miR-29a in the arcuate nucleus (Figure 2) ⇨ vi). POMC^Cre^-dependent knock-out of miR-29a (Figure 3, 4 and 6). In other words, we aimed to gradually reduce the number of confounding effects associated with microRNA loss in different ARH neuronal populations capable of regulating energy homeostasis [2]. The latter populations primarily include POMC and NPY neurons, within the melanocortin system, *Kiss/Tac2* neurons [9], vGlut2 neurons [36], tyrosine hydroxylase neurons [37], Ghrh neurons [38] and other cells. Notably, some NPY and *Kiss/Tac2* neurons originate from POMC^Cre^GFP + cells [8,9] and different populations of POMC and non-POMC neurons likely vary in the abundance of both miR-29 family microRNAs and their targets. As such, the POMC^Cre^miR-29CKO-associated phenotypes likely represent a combination of partially confounding and/or potentiating metabolic effects caused by different populations. However, the vast majority of POMC^Cre^GFP + cells continue expressing POMC throughout the adulthood [8] and the obesity phenotypes associated with POMC^Cre^-restricted PI3K-Akt-mTOR pathway over-activation are largely explained by inactivation of POMC neurons [[10], [11], [12]]. Thus, the POMC^Cre^miR-29CKO phenotypes described here might also be mostly contributed by POMC neurons, however this must be precisely demonstrated in the follow-up studies. Importantly, in this work we used multiple mutually exclusive experimental paradigms to demonstrate the *in vivo* phenotypes: males/females, chow/high fat diets, aged/young animals, control groups with identical genetotypes but treated with different viral vectors/non-transgenic littermates with identical viral transduction (S2 Table). This allowed us to prove the critical metabolic role of miR-29 in mature hypothalamic neurons derived from the POMC-expressing lineage. In some of the experiments, we additionally transduced POMC^Cre^miR-29CKO with rAAVs equipped with Cas9-targeted sgRs to restrain the possible off-target effects of sgRs (Figure 3, Figure S3). Such manipulations might have compromised the on-target efficiency and as such, modulated or blunted the phenotypes in these experiments. Nevertheless, the main rationale behind this approach was to use the most stringent conditions in the experimental design.

Notably, all members of the miR-29 family i). share high sequence similarity (Figure 1B) and hence an overlapping targetome (Table S3) while ii) being abundant in the mouse hypothalamus (Figure 1C). Hence, according to our *in vivo* experimental design, we POMC^Cre^-dependently deleted either one or both clusters of this family. Indeed, all family members were predicted and/or validated to target *Nras* (Table S3) and more generally PI3K-Akt-mTOR pathway (Figure 1A). Thus, in addition to miR-29a, miR-29b and miR-29c might also contribute to the Dicer-associated hypothalamic obesity with *Nras* being highly likely just one of the contributing targets. Interestingly, obesity in POMC^Cre^miR-29CKO mice was more pronounced in females in accordance with other reports about gender-biased metabolic phenotypes associated with the POMC^Cre^ transgenic line [39,40]. Furthermore, metabolic cage analyses revealed the decreased energy expenditure indicating that food intake is not the only driver of the obesity phenotype in these mice.

This is why we anticipate an involvement of other yet unidentified pathways and targets contributing to miR-29 family-mediated regulation of energy homeostasis by ARH neurons. Our study thus is just the first step towards identification of mechanisms controlling hyperphagia, energy expenditure reduction, insulin resistance and gender bias associated with miR-29 family knock-out, as well as towards answering the question whether the phenotypes described here are relevant in the human context. This work might stimulate the development of novel metabolic syndrome-targeting therapeutics. However, in a view of broad target specificity of microRNAs and therefore safety concerns, they should rather be aimed on critical targets instead of directly acting on this microRNA family.

## 4 Materials and methods

### 4.1 Animal models

All experimental procedures in mice of both genders were conducted on the C57BL/6N genetic background (at least nine backcrosses) at the German Cancer Research Center (DKFZ) for CamK^CreERT2^Dicer^fl/fl^ (also referred to as DicerCKO) mice [13] and in Shanghai Jiao Tong University for B6;129-Gt (ROSA)26Sor^tm1(CAG-cas9^^∗^^,−EGFP)Fezh^/J (JAX: 024857, further referred to as Cas9) mice [28] and Tg (Pomc1-cre)16Lowl/J (JAX: 005965, also referred to as POMC^Cre^) transgenic line [6] in accordance with international standards, as approved by the institutional and local authorities of Germany and China, respectively. Animals were maintained on a 12/12 h light/dark cycle with free access to water and standard chow food (3437, Kliba Nafag, and 1010088, Jiangsu XieTong, respectively) unless otherwise stated. High fat diet (60%) was from TrophicDiet (TP2330055B). Measurements of food intake and weight were performed on daily or weekly basis. Due to higher variability of initial body weights, weight data of males are represented in a normalized form throughout the manuscript. For metabolic profiling analysis, the animals were habituated to metabolic cages (Oxymax from Columbus instruments) for 4 days prior to metabolic data collection for the three subsequent days. Measurements of fat/lean mass body composition was performed on magnetic resonance imaging system MesoMR23-060H–I (NIUMAG, China) according to the manufacturer's protocol.

The mice were sacrificed during the light phase in a fed state and the collected tissues were used for post mortem analyses. Fat pads were weighed, fixed with 4% paraformaldehyde (PFA), dehydrated paraffin-embedded, sliced (thickness: 4 μm) and stained with hematoxylin & eosin according to a standard protocol. The images were acquired using a bright field microscope. The brains fixed with 4% PFA were vibratome-sliced (thickness: 40 μm) or prepared in O.C.T (SAKURA) and cryostat-sliced (Leica) (thickness: 20 μm). Alternatively, for RNA extraction, mice were anesthetized with avertin, and, after cervical dislocation, the brains were quickly removed and placed to cold PBS for microdissection. Spatula was used for dissection of the hypothalamus. Alternatively, for arcuate hypothalamic nucleus (ARH) microdissection, coronal brain sections were collected using a 0.5 mm mouse stainless steel brain matrix (RWD) slicing immediately after the optical chiasm and 2 mm rostrally from the first cut. Under microscope, ARH was microdissected from the resulting slice by two scalpels and snap-frozen to isolate total RNA for subsequent mRNA transcriptomics analyses.

### 4.2 Stereotaxic injections

Stereotaxic apparatus (Kopf Instruments or RWD) was used to bilaterally deliver the oligonucleotides or recombinant adeno-associated viral vectors (rAAVs) to ARH. Briefly, the mice were anesthetized by avertin (0.6 ml/25 kg) and their heads were fixed in the stereotaxic apparatus. The details of each experiment are described in Table S2.

Inactivation of *Dicer1* alleles in forebrain neurons of DicerCKO mice [13] was achieved by ten i.p. injections of 1 mg tamoxifen within 5 consecutive days in 8-week-old females as described in [13]. The effectivity of knockout in this model was previously validated [[24], [25], [26]]. Locked nucleic acids (LNA)-stabilized miR-29a-3p mimic or scrambled oligonucleotides (see also Figure 1 and Table S1) in cerebrospinal fluid (CSF) with 13.5% HiPerfect liposomal reagent (Qiagen) were administered on the 4th week after tamoxifen injections as described in [13]. Morphological and molecular analyses were done 6 weeks after tamoxifen injections.

To inactivate miR-29a/b1 cluster in ARH of the miR-29aCKO experimental group, Cas9 mice [28] were stereotaxically injected by a mixture of rAAVs, one of which was equipped with a double-sgRNA (sgR) cassette and mCherry reporter (File S2) and the other one expressed CAG synthetic promoter-driven Cre recombinase. For POMC^Cre^-specific knock-out experiments (referred to as POMC^Cre^miR-29aCKO, POMC^Cre^miR-29CKO experimental and POMC^Cre^miR-29CKO + NrasCKO groups), we bred Cas9 with POMC^Cre^ animals ensuring that POMC^Cre^ transgene is always heterozygous. Then, POMC^Cre^Cas9 mice were injected with rAAVs equipped with sgRNAs targeting mmu-miR-29a, -b1, -b2, -c, *Nras* and/or *Cas9* genes (referred to as sgR-2, -1, -3, -4, -n1, -n2, KamiCas9 and SliCES sgCas-c, respectively). No differences were detected for knock-out mice in Cas9 heterozygous or homozygous states for CAG^Cre^ or POMC^Cre^ experiments. See Tables S1, S2 and File S1 for full details of experiments and sgRNAs. For eight miR-29CKO experiments in the study, brains were fixed in 4% PFA and sliced for histology. Autofluorescence or immunofluorescence signals of GFP indicating mature POMC-lineage derived neurons and mCherry indicating cells infected by sgRNA-expressing rAAVs were visualized in the ARH during post-mortem analyses under the fluorescence stereomicroscope (Leica) to exclude mistargeted animals from the analyses. For all properly targeted miR-29CKO animals, 71.25% ± 9.86 demonstrated at least 120% weight gain.

### 4.3 Glucose metabolism

Clearance of injected glucose was measured by intraperitoneal glucose tolerance test. Briefly, mice were fasted for 6 h (Fig. S3G) or 16 h (all other experiments) followed by blood glucose monitoring prior to and after 2 g/kg body weight d-glucose injection. ACCU-CHEK active glucometer (Roche) at 15, 30, 45, 60, 90 and 120 min after injection.

### 4.4 Transcriptome analyses

Total RNA was obtained from the hypothalamus of wild type mice by mirVana microRNA Isolation kit (Ambion) or from ARH of mutants and controls by AllPrep Kit (QIAGEN) or RNeasy Mikro kit (Qiagen) or according to the manufacturer's protocol. The Mouse Sentrix-6 array mRNA profiling (Illumina) on ARH of animals injected with miR-29a-3p or scrambled LNA-modified mimics was performed according to the manufacturer's protocol (Table S4).

TaqMan qRT-PCR was used to detect mature microRNAs and performed according to the manufacturer's protocol (Thermo Fisher Scientific). Briefly, TaqMan MicroRNA Reverse Transcription Kit (Thermo Fisher Scientific) was used to synthesize cDNA followed by real-time PCR using TaqMan Universal Master Mix II (no UNG) kit (Thermo Fisher Scientific). TaqMan probes (Thermo Fisher Scientific) used in this work were used to detect mmu-miR-29a-3p, mmu-miR-29a-5p, mmu-miR-29b-3p, mmu-miR-29b-2-5p, mmu-miR-29b-5p, hsa-miR-29c-3p, mmu-miR-29c-5p, U6 (IDs: 002112, 002447, 000413,465008_mat, 002497, 000587, 001818 and 001973, respectively)

TB green qRT-PCR (Takara) was used to quantify expression of pri-miR-29a/b-1 and mRNAs (Table S1). Briefly, PrimeScript RT reagent kit with gDNA Eraser was used to synthesize cDNA followed by TB Green Premix Ex Taq II kit for qRT-PCR (Takara) according to the manufacturer's protocol. β-actin was used as a reference gene for expression normalization. The relative mRNA expression level was calculated as 2^-ΔΔCT^.

### 4.5 Immunofluorescent staining

Slides with cryosections were dried up at room temperature and then boiled in the boiling buffer (800 ml milli-Q water, 4 ml 1 M Tris pH8, 1.6 ml 0.5 EDTA) for antigen recovery. Then the brain sections were blocked in 150 μl blocking buffer (0.1 M PBS; 10% NGS and 0.1% Tween 20) and incubated overnight with the following primary antibodies: 1:300 Anti-Nras, Abcam, ab77392; 1/500 Anti-GFP, Proteintech, 50430-2-AP; 1/500 Anti-mCherry, Abclonal, AE002. Signals were visualized using the 1:400 diluted secondary antibody conjugated with AlexaFluor-488, -561 or −647 (Proteintech and Abclonal). After nucleic DNA staining by 4′,6-diamidino-2-phenylindole (DAPI, Sigma), slices were mounted with fluorescent anti-fade mounting medium (Dako). Images were acquired using the Ni-E A1 HD25 confocal microscope (Nikon).

### 4.6 *In situ* hybridization

For *in situ* hybridization, following 4% PFA perfusion, the brains were post-fixed with 4% PFA overnight, dehydrated in 30% (w/v) sucrose until they sunk to the bottom of tubes. Dehydrated tissues were then embedded in O.C.T (SAKURA) and sectioned (20 μm) by a cryostat (Leica). Frozen brain sections were air dried at room temperature under RNA free hood followed by overnight hybridization with digoxigenin-labelled miR-29a-3p probe (miRCURY LNA, QIAGEN, 339112YD00616795-BEF see Table S1) at 59 °C in the RNase-free incubator. Afterwards, the brain slices were washed and incubated overnight with 1:1500 anti-DIG-AP (Roche) antibody followed by BM-Purple (Roche) staining according to a standard procedure. Images were checked under bright field microscope. For co-localization studies immunofluorescent staining for GFP and mCherry was performed as described above and the images were acquired using the Ni-E A1 HD25 confocal microscope (Nikon).

### 4.7 *In silico* analyses

Single guide RNAs (sgRNAs, sgRs) were predicted and designed by an online tool CHOPCHOP (http://chopchop.cbu.uib.no/). The sgRNAs with highest scores and lowest predicted off-target sites were used for further validation (File S1).

For identification of potential targets, the following prediction tools/databases of validated interactions have been used: DIANA microT-CDS, GUUGle, MicroTar, miRanda, miRDB, miRmap, miRWalk, PITA, RNA22 V2, TargetScan, TarBase v.8, TargetSpy as indicated in Table S3. Pathway information was integrated from the Kyoto Encyclopedia of Genes and Genomes (KEGG, https://www.kegg.jp/).

3D structures of human (Uniprot: P01111) and mouse (Uniprot: P08556) Nras proteins were downloaded from Alphafold [33] Protein Structure database (https://alphafold.ebi.ac.uk/) and aligned in PyMol.

### 4.8 Cell culture

All cell lines were cultured at 37 °C in the incubator supplied with 5% of CO_2_ using DMEM basic medium (Gibco) with 10% FBS (Gibco) unless otherwise stated.

HeLa cells were reverse-transfected by antagomiRs (Ambion). Briefly, the OptiMEM supplemented with sucrose, lipofectamine 2000, gelatine and fibronectin (Thermo Fisher Scientific) were used to coat the plates prior to vacuum-centrifugation. The final concentration of each antagomiR was 121.3 fmol/μl. Insulin and rapamycin were used at concentrations 300 nM and 500 nM, respectively. At least three replicates per group were used. Cells were plated at a density 100,000/ml in 100% pyruvate-supplemented AIM V (Thermo Fisher Scientific) to limit the insulin-related effects. The S235/236-phosphorylated S6 ribosomal protein immunofluorescent signal was detected by a primary antibody (1:100 Cell Signaling, Cat# 4857S RRID: AB_2181035) followed by the secondary AlexaFluor-594-conjugated antibody (Invitrogen Cat# A11091 RRID: AB_1500116) in 9 images per well from at least 3 replicates.

For the dual-luciferase assay, 389 bases spanning the highly conserved putatively critical miR-29a target site of 3′-untranslated region (UTR) of *Nras* or the same region with the reverse sequence-mutated target site were subcloned into pmirGLO dual-luciferase plasmid (Promega). Double-stranded miR-29a-3p or negative control mimics (Genepharma) were transfected simultaneously with pmirGLO-Nras into HEK293T cells with the relation 30 pmol mimics per 1 μg plasmid DNA using lipofectamine2000 (Thermo Fisher Scientific) according to the manufacturer's protocol. Luminescence was induced by Dual-Luciferase Reporter Assay System kit (Promega, E1960), detected by the Synergy 2 luminometer (BioTek) and normalized by renilla luciferase signal followed by a standard analysis according to the manufacturer's protocol. See details about oligonucleotides used in this study in Table S1.

### 4.9 On-target efficiency of single guide RNAs

For the efficiency check, each sgRNA and the respective response sequence were subcloned into originally designed (manuscript in preparation) all-in-one split firefly luciferase plasmid equipped with sgRNA cassette under RNApol III promoter, SpCas9 and renilla luciferase for transfection control (see details in Table S1). A vector with the sgRNA subcloned but without a sgRNA response sequence was used as a negative control in subsequent analyses. Next, the vector was transfected to HEK293T cells using lipofectmine2000 (Thermo Fisher Scientific) according to the manufacturer's protocol. Luminescence was induced by Dual-Luciferase Reporter Assay System kit (Promega, E1960), detected by luminometer Synergy 2 (BioTek) and normalized by renilla luciferase signal followed by a standard analysis.

Additional validation of on-target activities of sgRNAs was done by measuring miR-29a-3p content by TaqMan in immortalized mouse hippocampal cell line HT-22 transfected by SpCas9-equipped HP180 vectors (a generous gift from Hui Yang, Table S1 and File S2) expressing sgR-1, sgR-2, sgR-3, sgR-4 targeting the miR-29 family (Figures 3C and 5E, see also File S2 for details of the subcloning of sgRs into HP180). The selected validated sgRs were used for subsequent off-target analysis (see below) and later subcloned (File S1) into a double-sgRNA cassette-vector equipped with mCherry fluorescent reporter protein (File S2) and then packaged into serotype 9 adeno-associated virus (rAAV). The titers and the amounts of rAAVs used for each experiment are indicated in Table S2).

Flow cytometer (BD, FACSAria II) was used to sort GFP positive HP180-transfected HT-22 cells for subsequent miR-29a-3p TaqMan qPCR (Figure 3C).

### 4.10 Off-target analyses

Based on the CHOPCHOP (http://chopchop.cbu.uib.no/) prediction results (Table S3), we identified all off-target loci within the mouse genome with up to 3 mismatches to verify *in vitro* and *in vivo*. Each sgRNA was subcloned into HP180 (a generous gift from Hui Yang, Files S1-2 and Table S1) and then the resulting construct was transfected into two different mouse cell lines with intact genomes: mouse fibroblast cells 3T3-L1 or immortalized mouse hippocampal cells HT-22.3T3-L1 cell line was transfected by the Lonza Nucleofector using its specific kit for undifferentiated 3T3-L1 cells, while HT-22 cell lines were transfected by lipofectamine2000 (Thermo Fisher Scientific) according to the manufacturer's protocol. The efficiency of transfection was estimated to be around 40%. Isolated total DNA was used for subsequent PCR.

For *in vivo* off target analyses, we used POMC^Cre^miR-29CKO + NrasCKO mice with a simultaneous knock-out of the miR-29 family and the *Nras* gene (Figure 6). Total DNA was isolated from microdissected ARH from nine mice and mixed to serve as a template for subsequent PCR.

Primer pairs were selected to span the putative off-target cutting site avoiding to locate it on the end of the PCR product (File S1). The primers were designed to include a barcode and protection bases for sequencing analyses. PCR was performed according to a standard protocol and the gel-extracted single bands of the expected lengths were used for next-generation sequencing (Illumina NovaSeq).

### 4.11 Statistical analysis

Data were analyzed by Pearson correlation test, Wald test, Student's two-tailed unpaired t-test, one- or two-way ANOVA followed by pairwise comparison between means adjusted by Holm–Sidak post-hoc test as indicated in figure legends. Metabolic profiling data were analyzed by ANCOVA with the body weight as a covariate. *p* values less than 0.05 were considered significant (∗, *p* < 0.05; ∗∗, *p* < 0.01; ∗∗∗, *p* < 0.001; ∗∗∗∗, *p* < 0.0001) with respect to control groups unless otherwise stated. Data in the figures and in the text are expressed as a mean ± standard error of mean (SEM). Analyses were performed using GraphPad Prism and National Mouse Metabolic Phenotype Center (https://www.mmpc.org/shared/regression.aspx) software.

### 4.12 Resource and data Availability

All unique/stable reagents and data generated in this study are available upon request from the Lead Contact without restriction.

## Funding

This work was financially supported by grants to IAV from the 10.13039/501100001809National Natural Science Foundation of China BC0800209, Foreign Non-Chinese Principal Investigator grant of Shanghai Jiao Tong University (SJTU) AF0800056 and Start-up package of SJTU WF220408008.

## Author contributions

Conceptualization: IAV; Data curation: YM, VS, RK, HE, WK, SSN, IAV, JR; Formal analysis: YM, NM, ZL, VS, RK, NK, SSN, IAV, JR, AT, AD; Funding acquisition: IAV; Investigation: YM, NM, YL, ZL, YX, XK, WK, SSN, IAV, JB, NB, HA, PMM; Methodology: YM, VS, HE, IAV, JR; Project administration: QL, SSN, IAV; Resources: HE, GS, VG, HY, TS; Software: VS, RK, JR; Supervision: IAV; Validation: WK, IAV; Visualization: YM, VS, NK, IAV, CG; Writing – original draft: YM, IAV; Writing – review & editing: YM, SSN, IAV.
