# Supplementary material for: Neuronal miR-29a protects from obesity in adult mice
Source: Mol Metab. 2022 Apr 29;61:101507. doi: 10.1016/j.molmet.2022.101507 (PMC9114687; doi:10.1016/j.molmet.2022.101507)
Supplement: Multimedia component 4 [file mmc4.pdf]

**Supplementary materials for**

# Neuronal miR-29a protects from obesity in adult mice

Yuan Ma, Nicola Murgia, Yu Liu, Zixuan Li, Chaweewan Sirakawin, Ruslan Konovalov, Nikolai Kovzel, Yang Xu, Xuejia Kang, Anshul Tiwari, Patrick Malonza Mwangi, Donglei Sun, Holger Erfle, Witold Konopka, Qingxuan Lai, Syeda Sadia Najam, Ilya A. Vinnikov

**This PDF file includes:**

Figures S1-S5

Tables S1-S3

Legends for Files S1-S3

**Other supplementary materials for this manuscript include the following:**

Files S1-S3

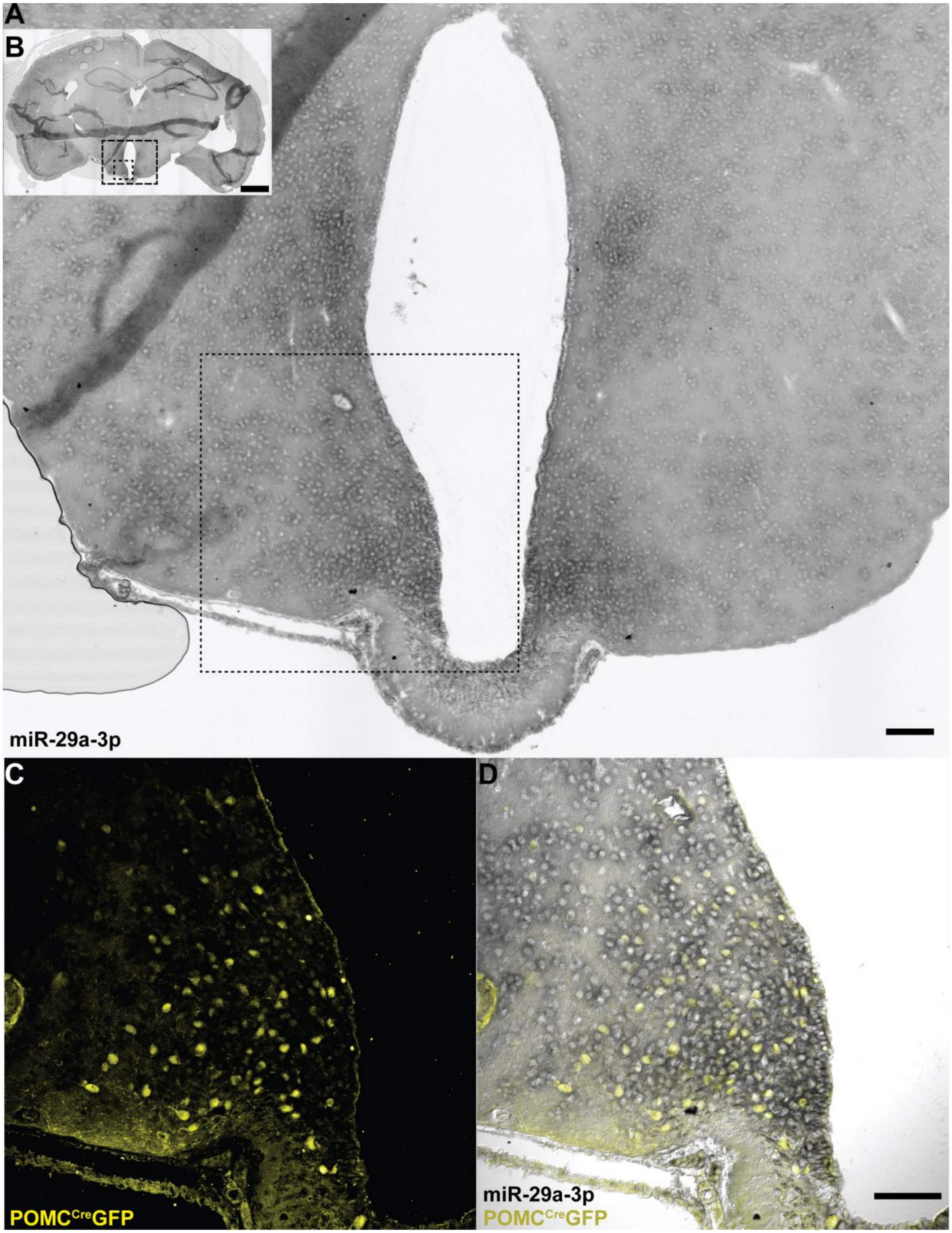


**Figure S1. Expression of miR-29a-3p in the adult mouse hypothalamus.** (**A**-**D**) Coronal brain section overview (**A**, **B**) and the arcuate hypothalamic nucleus microphotographs of miR-29a-3p *in situ* hybridization demonstrating its abundance in POMC$^{Cre}$GFP+ neurons and other cells within the ventral hypothalamus of adult POMC$^{Cre}$Cas9 mice. Scale bars (in µm): 100 (**A**, **C**, **D**), 1000 (**B**).

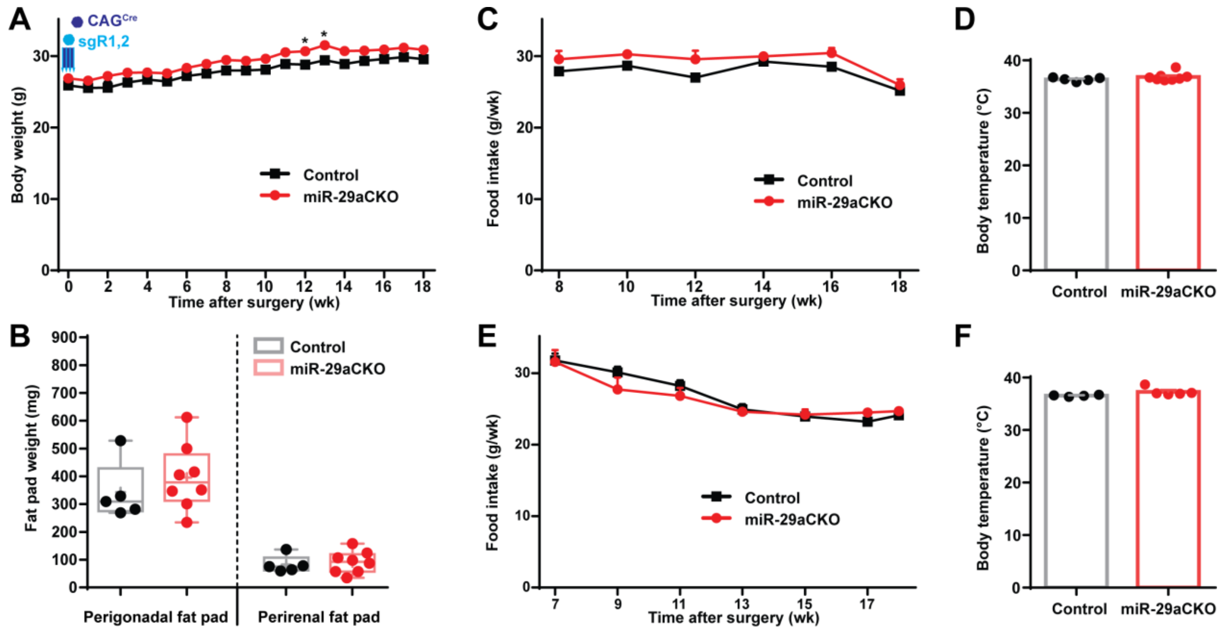


**Figure S2. Metabolic phenotypes in adult mice with a knock-out of miR-29 family in the arcuate hypothalamic nucleus**. (**A-F**) Phenotypic analyses of *in vivo* experiment to knock-out the miR-29a/b-1 cluster in the arcuate hypothalamic nucleus (ARH) of male (**A-D**) and female (**E**,**F**) adult miR-29aCKO mice. Bilateral injections of sgRNA (sgR)- and CAG-Cre-equipped adeno-associated viral vectors (rAAVs) into 2 coronal planes (see **S1 Table**) of adult Cas9 mice are indicated by 4 arrows on the week 0 (**A**). Male mice were analyzed for body (**A**) and perigonadal (**left**)/perirenal (**right**) fat pad (**B**) weights. In addition to these parameters, food intake and rectal temperature were analyzed in male (**C**,**D**) and female (**E**,**F**) miR-29aCKO mice with the cluster deletion in the arcuate hypothalamic nucleus or control littermates (n= 8 and 5 for (**A,B,C,D**), 5 and 7 for (**E,F**), respectively). Error bars represent SEM. *, $p < 0.05$ as assessed by 2-way ANOVA followed by post-hoc Holm-Sidak pairwise comparison tests.

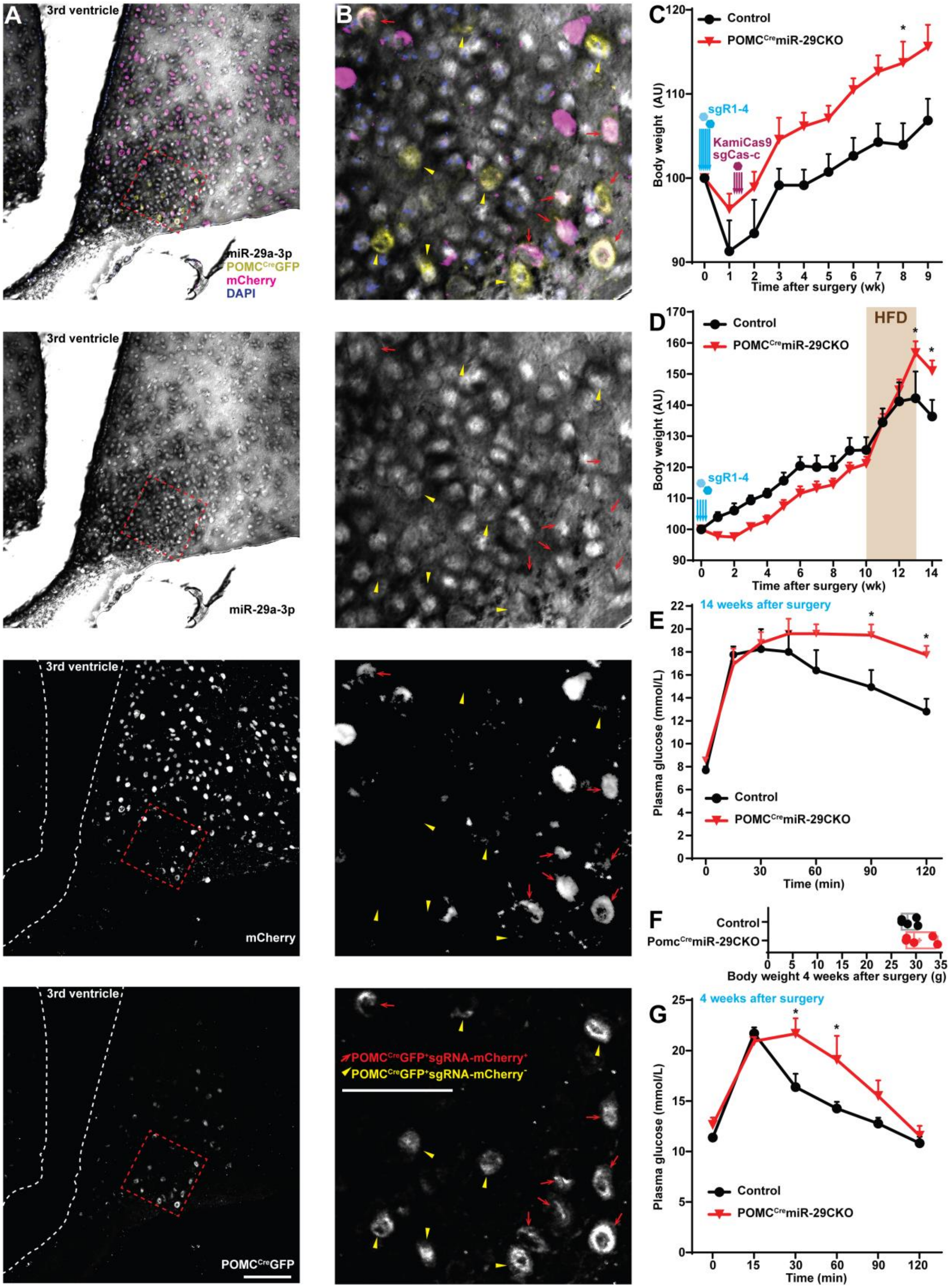


**Figure S3. Molecular and phenotypic analyses of POMC$^{\mathrm{Cre}}$miR-29aCKO mice.** (**A**-**B**) Overview (**A**) and zoomed-in (**B**) microphotographs of the arcuate hypothalamic nucleus (ARH) demonstrating co-localization of miR-29a-3p *in situ* hybridization with POMC$^{\mathrm{Cre}}$Cas9-GFP and mCherry labelling the cells infected with double-sgR cassette-equipped rAAV vectors

in adult POMC$^{Cre}$miR-29CKO mice. The zoomed-in region is outlined by dashed red square. Mature POMC$^{Cre}$GFP neurons transduced (POMC$^{Cre}$GFP$^{+}$mCherry$^{+}$ cells) or not transduced (POMC$^{Cre}$GFP$^{+}$mCherry$^{-}$ cells) by rAAV equipped with the sgRs targeting both miR-29 clusters, are indicated by red arrows and yellow triangles, respectively. (**C**) Body weight dynamics in adult POMC$^{Cre}$miR-29CKO and control male mice following the infection of their ARH with rAAVs expressing sgR1-4 at the age of 21 weeks (n = 6 and 7, respectively). 10 days later, all animals received rAAVs expressing Cas9-targeting sgRs. (**D**,**E**) Body weight dynamics (**D**) and glucose tolerance test 14 weeks after surgery (**E**) in young male POMC$^{Cre}$miR-29CKO and control mice (n = 7 and 5, respectively) stereotaxically injected to ARH with rAAVs equipped with sgR1-4 targeting all microRNAs in the miR-29 family. High fat diet treatment was applied from the 10$^{th}$ to the 13$^{rd}$ week. (**F**,**G**) Body weight (**F**) and glucose tolerance (**F**) analyses 4 weeks after surgery in young POMC$^{Cre}$miR-29CKO and control males (n = 5). Error bars represent SEM. *, $p < 0.05$ as assessed by 2-way ANOVA followed by post-hoc Holm-Sidak pairwise comparison test. Scale bars (in μm): 100 (**A**), 50 (**B**).

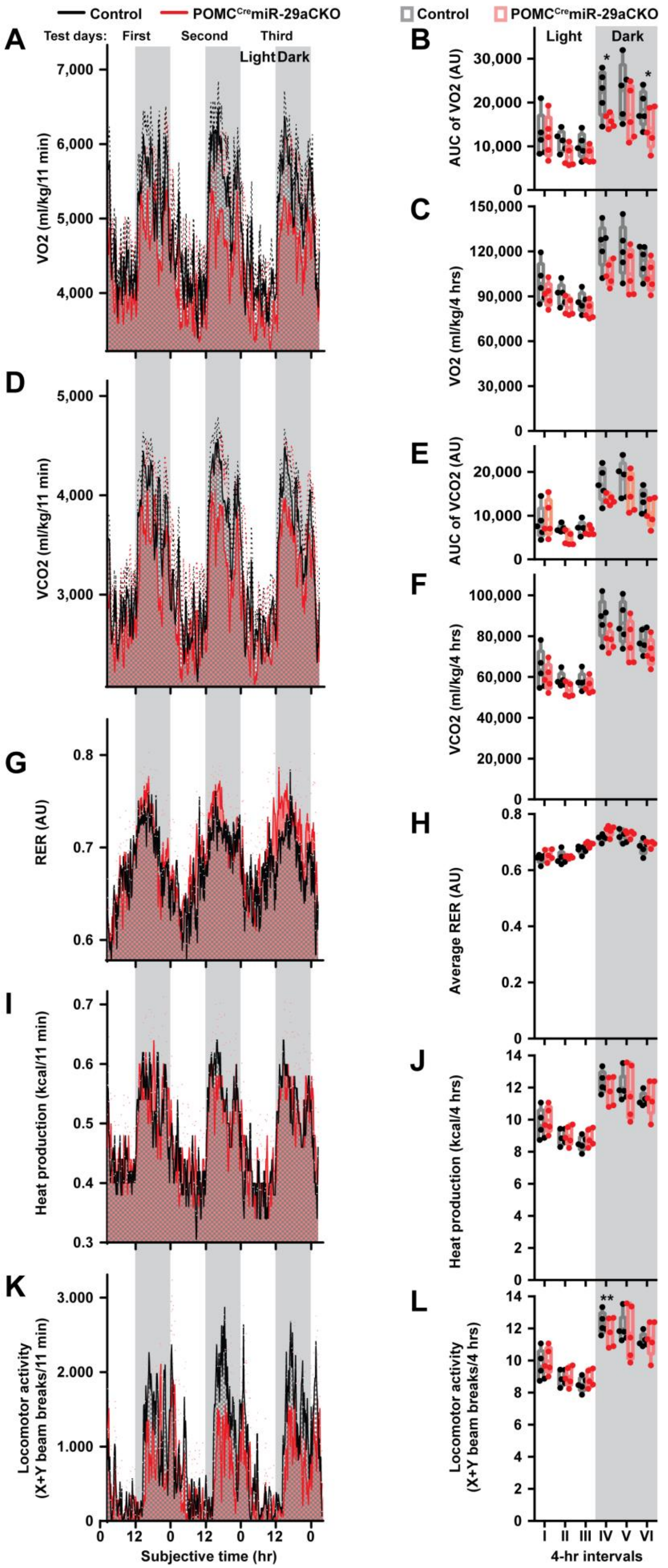


**Figure S4. Metabolic profiling of adult POMC$^{Cre}$miR-29aCKO mice.** (**A**-**L**) Raw data of 3 consequent testing days (**A**,**D**,**G**,**I**,**K**) and ANCOVA quantification in 4 hr-intervals of the 3-day averages in POMC$^{Cre}$miR-29aCKO mice (n=5) five weeks after surgery (**B**,**C**,**E**,**F**,**H**,**J**,**L**). Tested parameters included cumulative oxygen consumption (**A**-**C**) and carbon dioxide production (**D**-**F**), average RER (**G**,**H**), cumulative heat production (**I**,**J**) and locomotor activity (**K**,**L**). Areas under the curves (AUC, white-grey and pink-grey hatching in the raw data for control and experimental groups, respectively) were quantified for oxygen consumption and carbon dioxide production (**B** and **E**, respectively). Error bars in the raw data (**A**,**D**,**G**,**I**,**K**) are presented as dashed curves above the mean value curves. Error bars represent SEM. *, $p < 0.05$, **, $p < 0.01$ as assessed by ANCOVA analysis with body mass used as a covariate.

**Figure S5**. **Nras is a target of miR-29a-3p.** (**A**) Euler diagram representing the intersection of the members of the Kyoto Encyclopedia of Genes and Genomes PI3K-Akt pathway (**yellow**) with genes significantly down-regulated in the arcuate hypothalamic nucleus of DicerCKO mice upon delivery of miR-29a-3p mimics compared to the DicerCKO-Scrambled group (**pink**) and 9 genes including *Nras* yielded from the intersection. (**B**) Nras protein structure is highly conserved between mice and humans. Cartoon representation of superimposed 3D structures from Alphafold (26) Protein Structure database for mouse (**yellow**) and human (**cyan**) Nras proteins. Echo-

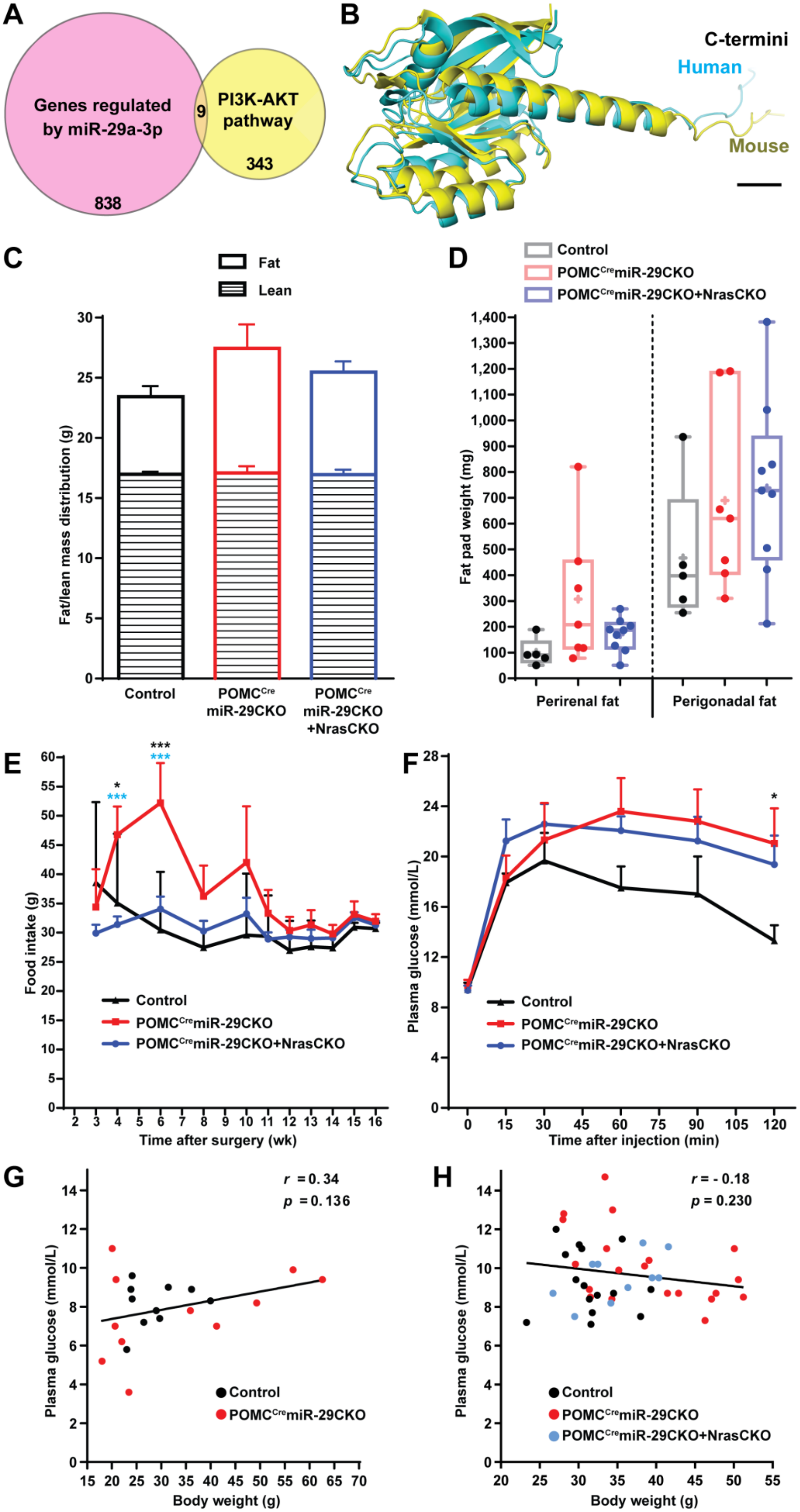

MRI-based body composition analysis (**C**), perirenal (**left**)/perigonadal (**right**) fat pad weights (**D**), food intake (**E**) and glucose tolerance test 19 weeks after surgery (**F**) in adult POMC$^{Cre}$miR-29CKO, POMC$^{Cre}$miR-29CKO+NrasCKO or control males (n= 7, 9, 5 respectively). (**G**, **H**) Pierson correlation analysis of fasting glucose and weight at the time of analysis for females (**G**, n = 11,10 for Control and POMC$^{Cre}$miR-29CKO, respectively) and males (**H**, n = 15,10,11 for Control, POMC$^{Cre}$miR-29CKO and POMC$^{Cre}$miR-29CKO+NrasCKO, respectively) in all glucose tolerance tests in this study. Error bars represent SEM. *, $p < 0.05$; ***, $p < 0.001$ as assessed by 2-way ANOVA followed by post-hoc Holm-Sidak pairwise comparison tests vs. the groups outlined with a respective color. Scale bar (in nm): 10.

**Table S1.** (relates to **Fig. 1-6**) **Oligonucleotides used in this work.**

| **Oligonu-cleotides** | **Antisense strand or forward primer*** | **Sense strand or reverse primer*** | **Company** |
|---|---|---|---|
| | **Reverse-transfection of antagomiRs to HeLa cells (Fig. 1A)** | | |
| miR-15a-5p-inh | uagcagcacauaaugguuugug | | Ambion |
| miR-15b-5p-inh | uagcagcacaucaugguuuaca | | Ambion |
| miR-26a-5p-inh | uucaaguaauccaggauaggcu | | Ambion |
| miR-26b-5p-inh | uucaaguaauucaggauaggu | | Ambion |
| miR-27a-3p-inh | uucacaguggcuaaguuccgc | | Ambion |
| miR-27b-3p-inh | uucacaguggcuaaguucugc | | Ambion |
| miR-29a-3p-inh | uagcaccauuugaaaucgguua | | Ambion |
| miR-29b-3p-inh | uagcaccauuugaaaucaguguu | | Ambion |
| miR-29c-3p-inh | uagcaccauuugaaaucgguua | | Ambion |
| miR-93-5p-inh | caaagugcuguucgugcagguag | | Ambion |
| miR-103a-3p-inh | agcagcauuguacagggcuauga | | Ambion |
| miR-103b-inh | ucauagcccuguacaaugcugcu | | Ambion |
| miR-107-inh | agcagcauuguacagggcuauca | | Ambion |
| miR-206-inh | uggaauguaaggaagugugugg | | Ambion |
| miR-320-3p-inh | aaaagcugggguugagagggcga | | Ambion |
| miR-320b-inh | aaaagcuggguugagagggcaa | | Ambion |
| miR-320c-inh | aaaagcuggguugagagggu | | Ambion |
| miR-320d-inh | aaaagcuggguugagagga | | Ambion |
| | **In situ probes (Fig. S1, S3)** | | |
| miR-29a-3p-insitu | TAACCGATTTCAGATGGTGCT–Digoxigenin | | Qiagen |
| | **Injection of LNA-modified microRNA-mimics *in vivo* (Fig. 1E-G)** | | |
| 29a-3p-L | uagcaccaucugaaaucgguTT | AccgauuucagauggugcuaTT | Exiqon |
| Scr-L | ugggcguauagacguguuacacTT | GuguaacacgucuauacgcccaTT | Exiqon |
| | **Transfection of microRNA mimics *in vitro* (Fig. 5D-F)** | | |
| 29a-3p | accgauuucagauggugcuauu | uagcaccaucugaaaucgguua | Genepharma |
| NC | acgugacacguucggagaaTT | uucuccgaacgugucacguTT | Genepharma |
| | **Primers for qRT-PCR (Fig. 2D, 5F)** | | |
| pri-miR29a/b | ACCCCTTAGAGGATGACTGATTTC | TCCCCAATCATTATAACCGATTTCA | Genewiz |
| Nras | ACTGAGTACAAACTGGTGGTGG | TCGGTAAGAATCCTCTATGGTGG | Genewiz |
| β-actin | GGCTGTATTCCCCTCCATCG | CCAGTTGGTAACAATGCCATGT | Genewiz |

**Table S1**. (cont.)

**Subcloning of sgRNA response sequences to a split-luciferase Cas9-equipped vector (Fig. 2B, 3B, 6C)**

| | | | |
|---|---|---|---|
| sgR-2-NR | CGCGTCATTATAACCGATTTCAGATGGT GCA | CCAATATTTACGTGCTGCTA GAG | Genewiz |
| sgR-1-NR | CGCGAGGAAGCTGGTTTCATATGGTGGT GCA | CCACCATATGAAACCAGCTT CCT | Genewiz |
| sgR-3-NR | CGCGTGGAAGCTGGTTTCACATGGTGGT GCA | CCACCATGTGAAACCAGCTT CCA | Genewiz |
| sgR-4-NR | CGCGTGTCTAGCACCATTTGAAATCGGT GCA | CCGATTTCAAATGGTGCTAG ACA | Genewiz |
| sgR-n1-NR | CGCGAAACTGGTGGTGGTTGGAGCCGGT GCA | CCGGCTCCAACCACCACCAG TTT | Genewiz |
| sgR-n2-NR | CGCGCTTCGCCTGTCCTCATGTACCGGT GCA | CCGGTACATGAGGACAGGCG AAG | Genewiz |

**Subcloning of sgRNAs to a split-luciferase Cas9-equipped vector (Fig. 2B, 3B, 6C)**

| | | | |
|---|---|---|---|
| sgR-2-NSG | CACCGTCATTATAACCGATTTCAGA | AAACTCTGAAATCGGTTATAATGAC | Genewiz |
| sgR-1-NSG | CACCGAGGAAGCTGGTTTCATATGG | AAACCCATATGAAACCAGCTTCCTC | Genewiz |
| sgR-3-NSG | CACCGTGGAAGCTGGTTTCACATGG | AAACCCATGTGAAACCAGCTTCCAC | Genewiz |
| sgR-4-NSG | CACCGTGTCTAGCACCATTTGAAAT | AAACATTTCAAATGGTGCTAGACAC | Genewiz |
| sgR-n1-NSG | CACCGAAACTGGTGGTGGTTGGAGC | AAACGCTCCAACCACCACCAGTTTC | Genewiz |
| sgR-n2-NSG | CACCGCTTCGCCTGTCCTCATGTAC | AAACGTACATGAGGACAGGCGAAGC | Genewiz |

**Subcloning of sgRNAs to double-sgRNA cassette-equipped rAAV vector (Fig. 2-4, 6, S2-S5)**

| | | | |
|---|---|---|---|
| sgR-2-A | ACCGTCATTATAACCGATTTCAGA | AACTCTGAAATCGGTTATAATGAC | Genewiz |
| sgR-1-A | GAGGAAGCTGGTTTCATATGGGTTTT | CCATATGAAACCAGCTTCCTCGGGAA | Genewiz |
| sgR-3-A | ACCGTGGAAGCTGGTTTCACATGG | AACCCATGTGAAACCAGCTTCCAC | Genewiz |
| sgR-4-A | GTGTCTAGCACCATTTGAAATGTTTT | ATTTCAAATGGTGCTAGACACGGGAA | Genewiz |
| sgR-n1-A | ACCGAAACTGGTGGTGGTTGGAGC | AACGCTCCAACCACCACCAGTTTC | Genewiz |
| sgR-n2-A | GCTTCGCCTGTCCTCATGTACGTTTT | GTACATGAGGACAGGCGAAGCGGGAA | Genewiz |
| KamiCas9-A | ACCGAATGGAGTACTTCTTGTCCA | AACTGGACAAGAAGTACTCCATTC | Genewiz |
| sgCas-c-A | GGGCTACGCCGGCTACATTGAGTTTT | TCAATGTAGCCGGCGTAGCCCGGGAA | Genewiz |

**Subcloning of sgRNAs to Cas9-equipped vector HP180 (Fig. 3C, 5E-F)**

| | | | |
|---|---|---|---|
| sgR-2-H | CACCGTCATTATAACCGATTTCAGA | AAACTCTGAAATCGGTTATAATGAC | Genewiz |
| sgR-1-H | CACCGAGGAAGCTGGTTTCATATGG | AAACCCATATGAAACCAGCTTCCTC | Genewiz |
| sgR-3-H | CACCGTGGAAGCTGGTTTCACATGG | AAACCCATGTGAAACCAGCTTCCAC | Genewiz |
| sgR-3-H | CACCGTGTCTAGCACCATTTGAAAT | AAACATTTCAAATGGTGCTAGACAC | Genewiz |
| sgR-n1-H | CACCGAAACTGGTGGTGGTTGGAGC | AAACGCTCCAACCACCACCAGTTTC | Genewiz |
| sgR-n2-H | CACCGCTTCGCCTGTCCTCATGTAC | AAACGTACATGAGGACAGGCGAAGC | Genewiz |

**In-fusion subcloning of Nras 3’-UTR into the dual-luciferase vector from Promega (Fig. 5D)**

| | | | |
|---|---|---|---|
| Nras-luc | CTAGTTGTTTAAACGGCCCTCAGT TCCCTTCTCAGC | TAGACTCGAGGCTAGGGCTTTTCGA GACAGGGTTTCTC | Genewiz |

* RNA nucleotides are indicated with small letters, DNA nucleotides—with capital letters, LNA-modified oligonucleotides are shown in red.

**Table S2.** (relates to **Fig. 1-4**) **Details about bilateral stereotaxic injections performed in this work.**

| **Details of the experimental groups*** | **Details of the control groups*** | **Coordinates#, correlations¶ and figure references** |
|---|---|---|
| 13 wk-old CamK$^{CreERT2+}$Dicer$^{fl/fl}$ females injected with 0.5 µl per site of miR-29a mimics 4 weeks after tamoxifen injections. | 13 wk-old CamK$^{CreERT2+}$Dicer$^{fl/fl}$ (DicerCKO-Scrambled group) and 11 wk-old CamK$^{CreERT2-}$Dicer$^{fl/fl}$ (Control-Scrambled group) females injected with 0.5 µl per site of scrambled oligonucleotides 4 weeks after tamoxifen injections. | -1.46; ±0.25; -5.75<br>*r*/*p* for correlations of weight gain vs. age and initial weights: 0.35/0.149 and 0.41/0.091<br>(**Fig. 1**) |
| 12 wk-old Cas9$^{+/-}$ males injected with 0.2 µl per site of 1:1 (vol/vol) of rAAVs equipped with sgR-1/2 (titer in vg/ml: 9.7 *10$^{13}$) and CAG-Cre (10$^{10}$). | 12 wk-old Cas9+/wt males injected with 0.2 µl per site of rAAV lacking the sgR cassette (1.35 *10$^{14}$). | -1.46, ±0.2, -5.8<br>-0.18/0.545 and 0.46/0.118<br>(**Fig. S2A-D**) |
| 9 wk-old Cas9$^{+/-}$ females injected with 0.2 µl per site of 1:1 (vol/vol) of rAAVs equipped with sgR-1/2 (titer: 9.7 *10$^{13}$) and CAG-Cre (10$^{10}$). | 13 wk-old Cas9+/wt females injected with 0.2 µl per site of rAAV lacking the sgR cassette (1.35 *10$^{14}$). | -1.46, ±0.2, -5.8<br>−0.56/0.119 and 0.51/0.165<br>(**Fig. 2E-G, S2E-G**) |
| 24 wk-old POMC$^{Cre+}$Cas9$^{+/+}$ females injected to 6 coordinates with 0.3 µl per site of 1:1 of rAAVs equipped with sgR-1/2 (9.7 *10$^{13}$) and sgR-3/4 (4.86 *10$^{13}$).<br>1 week later, 0.3 µl per site of rAAV equipped with KamiCas9 and SliCES sgCas-c were injected to 4 coordinates (2.66 *10$^{13}$). | 23 wk-old POMC$^{Cre-}$Cas9$^{+}$ females injected with 0.3 µl per site of empty sgR cassette-equipped rAAV (2.37 *10$^{13}$).<br>1 wk later, 0.3 µl per site of KamiCas9/SliCES sgCas-c rAAV vector was injected to 4 coordinates (2.66 *10$^{13}$). | -1.46, ±0.2, -5.8<br>-2.3, ±0.2, -5.5<br>-1.82, ±0.25, -5.75<br>-1.46, ±0.2, -5.8<br>-2.3, ±0.2, -5.5<br>0.09/0.814 and 0.12/0.741<br>(**Fig. 3E-G**) |
| 14 wk-old POMC$^{Cre+}$Cas9$^{+/-}$ females injected with 0.3 µl per site of sgR-1/2-equipped rAAV (9.7 *10$^{13}$). | 16 wk-old POMC$^{Cre+}$Cas9$^{+}$ females injected with 0.3 µl per site of empty sgR cassette-equipped rAAV (1.35*10$^{14}$). | -1.46, ±0.2, -5.8<br>-2.3, ±0.2, -5.5<br>-0.44/0.170 and -0.04/0.913<br>(**Fig. 4A-I**) |
| 10 wk-old POMC$^{Cre+}$Cas9$^{+/+}$ females injected with 0.2 µl per site of sgR-1/2-equipped rAAV (9.7 *10$^{13}$). | 9 wk-old POMC$^{Cre-}$Cas9$^{+/+}$ females injected with 0.2 µl per site of sgR-1/2-equipped rAAV (2.37 *10$^{13}$). | -1.46, ±0.2, -5.8<br>-2.3, ±0.2, -5.5<br>0.18/0.645 and -0.54/0.133<br>(**Fig. S4A-Q**) |

**Table S2**. (cont.)

| | | |
|---|---|---|
| 11 wk-old POMC$^{Cre+}$Cas9$^{+}$ males injected with 0.2 µl per site of 1:1:1 of rAAVs equipped with sgR-1/2 (9.7 *10$^{13}$), sgR-3/4 (4.86 *10$^{13}$) and sgR-n1/2 (1.73 *10$^{13}$). An additional group received the same mixture but with the equivalent volume of PBS instead of the sgR-n1/2 vector. | 11 wk-old POMC$^{Cre+}$Cas9$^{+}$ males injected with 0.2 µl per site of empty sgR cassette-equipped rAAV (2.37 *10$^{13}$). | -1.46, ±0.2, -5.8<br>-2.3, ±0.2, -5.5<br>-0.38/0.065 and<br>-0.48/0.018<br>(**Fig. 6, S5C-F**) |
| 21 wk-old POMC$^{Cre+}$Cas9$^{+}$ males injected to 6 coordinates with 0.2 µl per site of 1:1 of rAAVs equipped with sgR-1/2 (9.7 *10$^{13}$) and sgR-3/4 (4.86 *10$^{13}$).<br>10 days later, 0.2 µl per site of rAAV equipped with KamiCas9 and SliCES sgCas-c were injected to 4 coordinates (2.66 *10$^{13}$). | 21 wk-old POMC$^{Cre-}$Cas9$^{+/+}$ males injected with 0.2 µl per site of 1:1 of rAAVs equipped with sgR-1/2 (9.7 *10$^{13}$) and sgR-3/4 (4.86 *10$^{13}$).<br>10 days later, 0.2 µl per site of rAAV equipped with KamiCas9 and SliCES sgCas-c were injected (2.66 *10$^{13}$). | 1.46, ±0.2, -5.8<br>-2.3, ±0.2, -5.5<br>-1.82, ±0.25, -5.75<br><br>-1.46, ±0.2, -5.8<br>-2.3, ±0.2, -5.5<br>0.02/0.938 and<br>-0.15/0.591<br>(**Fig. S3C**) |
| 10 wk-old POMC$^{Cre+}$Cas9$^{+/+}$ males injected with 0.2 µl per site of 1:1 of rAAVs equipped with sgR-1/2 (9.7 *10$^{13}$) and sgR-3/4 (4.86 *10$^{13}$). | 10 wk-old POMC$^{Cre-}$Cas9$^{-/-}$ males injected with 0.2 µl per site of rAAV lacking the sgR cassette (2.37 *10$^{13}$). | -1.46, ±0.2, -5.8<br>-2.3, ±0.2, -5.5<br>0.29/0.389 and<br>0.53/0.097<br>(**Fig. S3D-E**) |
| 10 wk-old POMC$^{Cre+}$Cas9$^{+/+}$ males injected with 0.3 µl per site of 1:1 of rAAVs equipped with sgR-1/2 (9.7 *10$^{13}$) and sgR-3/4 (4.86 *10$^{13}$). | 10 wk-old POMC$^{Cre-}$Cas9$^{+/+}$ males injected with 0.3 µl per site of 1:1 of rAAVs equipped with sgR-1/2 (9.7 *10$^{13}$) and sgR-3/4 (4.86 *10$^{13}$). | -1.46, ±0.2, -5.8<br>-2.3, ±0.2, -5.5<br>0.35/0.326 and<br>-0.43/0.213<br>(**Fig. 3F-G**) |

[*] Including sex, genotypes, injection volumes per site, titers of rAAVs (in vg/mL). [#] relative to Bregma (mm): A/P, anterio-posterior; M/L, medio-lateral; D/V, dorso-ventral. [¶] Weight gain (relative to initial weights) Pearson correlations (*r* and *p* values) with age and initial weight are indicated. rAAVs, recombinant adeno-associated viral vectors; vg/ml, vector genome copies per mL; sgR, sgRNA, single guide RNA.

**Table S3.** (relates to **Fig. 4**) **Tools and databases identifying Nras as a target of the miR-29 family in humans and mice**.

| Tool \ MicroRNA | miR-29a-3p (human/ mouse) * | miR-29b-3p (human/ mouse) | miR-29c-3p (human/ mouse) | Link |
|---|---|---|---|---|
| **DIANA microT-CDS** | ☑/☑ | ☑/☑ | ☑/☑ | http://diana.imis.athena-innovation.gr/DianaTools/index.php?r=microT_CDS/index |
| **GUUGle** | ☑/☑ | ☑/☑ | ☑/☑ | https://bibiserv.cebitec.uni-bielefeld.de/guugle |
| **MicroTar** | ☒/☑ | ☒/☒ | ☒/☑ | http://tiger.dbs.nus.edu.sg/microtar/ |
| **miRanda** | ☑/☑ | ☑/☑ | ☑/☑ | https://tools4mirs.org/software/target_prediction/miranda/ |
| **miRDB** | ☑/☑ | ☑/☑ | ☑/☑ | http://mirdb.org/ |
| **miRmap** | ☑/☑ | ☑/☑ | ☑/☑ | https://mirmap.ezlab.org/ |
| **miRWalk** | ☑/☑ | ☑/☒ | ☑/☑ | http://mirwalk.umm.uni-heidelberg.de/ |
| **PITA** | ☑/☑ | ☑/☑ | ☑/☑ | http://genie.weizmann.ac.il/pubs/mir07/mir07_data.html |
| **RNA22 V2** | ☑/☑ | ☑/☑ | ☑/☑ | https://cm.jefferson.edu/rna22/Interactive/ |
| **TargetScan** | ☑/☑ | ☑/☑ | ☑/☑ | http://www.targetscan.org/vert_71/ |
| **TarBase v.8** | ☑/☑ | ☑/☑ | ☑/☑ | http://carolina.imis.athena-innovation.gr/diana_tools/web/index.php?r=tarbasev8%2Findex |
| **TargetSpy** | ☑/☑ | ☑/☒ | ☑/☒ | http://webclu.bio.wzw.tum.de/targetspy/index.php?search=true |

* ☑, targeting of Nras was predicted by the tool; ☒, targeting of Nras was not predicted by the tool.

**File S1**. (separate file File S1.xlsx, relates to **Fig. 2-6**) **Single guide RNAs used in this study.**

**File S2**. (separate file File S2.docx, relates to **Fig. 2-6**) **Sequences of the constructs used in this work**.

**File S3**. (separate file File S3.xlsx, relates to **Fig. 1, 6**) **Microarray transcriptomics analysis of the arcuate hypothalamic nucleus**.
